# Reactive Oxygen Species-Inducible ECF σ Factors of *Bradyrhizobium japonicum*

**DOI:** 10.1371/journal.pone.0043421

**Published:** 2012-08-16

**Authors:** Nadezda Masloboeva, Luzia Reutimann, Philipp Stiefel, Rainer Follador, Nadja Leimer, Hauke Hennecke, Socorro Mesa, Hans-Martin Fischer

**Affiliations:** ETH, Institute of Microbiology, Zurich, Switzerland; Vrije Universiteit Brussel, Belgium

## Abstract

Extracytoplasmic function (ECF) σ factors control the transcription of genes involved in different cellular functions, such as stress responses, metal homeostasis, virulence-related traits, and cell envelope structure. The genome of *Bradyrhizobium japonicum*, the nitrogen-fixing soybean endosymbiont, encodes 17 putative ECF σ factors belonging to nine different ECF σ factor families. The genes for two of them, *ecfQ* (bll1028) and *ecfF* (blr3038), are highly induced in response to the reactive oxygen species hydrogen peroxide (H_2_O_2_) and singlet oxygen (^1^O_2_). The *ecfF* gene is followed by the predicted anti-σ factor gene *osrA* (blr3039). Mutants lacking EcfQ, EcfF plus OsrA, OsrA alone, or both σ factors plus OsrA were phenotypically characterized. While the symbiotic properties of all mutants were indistinguishable from the wild type, they showed increased sensitivity to singlet oxygen under free-living conditions. Possible target genes of EcfQ and EcfF were determined by microarray analyses, and candidate genes were compared with the H_2_O_2_-responsive regulon. These experiments disclosed that the two σ factors control rather small and, for the most part, distinct sets of genes, with about half of the genes representing 13% of the members of H_2_O_2_-responsive regulon. To get more insight into transcriptional regulation of both σ factors, the 5′ ends of *ecfQ* and *ecfF* mRNA were determined. The presence of conserved sequence motifs in the promoter region of *ecfQ* and genes encoding EcfQ-like σ factors in related α-proteobacteria suggests regulation via a yet unknown transcription factor. By contrast, we have evidence that *ecfF* is autoregulated by transcription from an EcfF-dependent consensus promoter, and its product is negatively regulated via protein-protein interaction with OsrA. Conserved cysteine residues 129 and 179 of OsrA are required for normal function of OsrA. Cysteine 179 is essential for release of EcfF from an EcfF-OsrA complex upon H_2_O_2_ stress while cysteine 129 is possibly needed for EcfF-OsrA interaction.

## Introduction

Extracytoplasmic function (ECF) σ factors are alternative bacterial RNA polymerase σ factors that play key roles in the response and adaptation of bacteria to different stresses and environments (for reviews and a comprehensive classification, see [1], [2]). ECF σ factors are members of the σ^70^ family, which is divided into four groups. Primary σ factors of group 1, to which the housekeeping σ factors belong, contain four conserved domains 1 to 4 and some of them also comprise an additional non-conserved region. They usually recognize promoters with the sequence TTGaca (−35) and TAtaaT (−10) [3]. In contrast, ECF σ factors belong to group 4 of the σ^70^ family and contain only the conserved domains 2 and 4. Many of them are thought to respond to environmental signals, they are often associated with an anti-σ factor, and usually auto-regulate their own expression [1], [2], [4]. Among the environmental cues are reactive oxygen species (ROS) which almost all bacteria encounter and against which even anaerobes have evolved defense mechanisms [5]–[7]. In aerobic organisms, ROS are generated also endogenously, e.g. by incomplete reduction of oxygen during respiration. The term ROS is generic, embracing not only free radicals such as superoxide anion (O_2_^−^) and hydroxyl radicals (OH^•^) but also hydrogen peroxide (H_2_O_2_) and singlet oxygen (^1^O_2_) (for reviews, see 8,9).

Generation of ROS occurs via different routes (for reviews, see [10]–[12]). Briefly, the best studied enzymatic generation of superoxide, and consequently hydrogen peroxide, originates from NADPH oxidases that catalyze the production of superoxide by the one-electron reduction of molecular oxygen using NADPH as an electron donor [11], [13]. The main source of singlet oxygen is the photosynthetic apparatus where it is generated in photosystem II as a side product by energy transfer from excited triplet-state chlorophyll pigments to O_2_ [14]. Energy can also be transferred to molecular oxygen by excited photosensitizers such as phytoalexins which are produced by plants in response to pathogens [15]. Apart from plant-derived sources, singlet oxygen is also produced in natural waters by the exposure of chromophoric dissolved organic matter to light [16].

Several ECF σ factors have been described to play a role in the response of bacteria to oxidative stress. Examples are *Streptomyces coelicolor* SigR which responds to disulfide stress produced by superoxide and diamide [17], *Caulobacter crescentus* SigT which is necessary for survival under osmotic and oxidative stress [18], and SigF of the same organism mediating the response to oxidative stress in stationary phase [19]. In the photosynthetic bacterium *Rhodobacter sphaeroides*, transcription of *rpoE* is increased upon singlet oxygen stress ( [20]; for review, see [21]) while RpoE activity is controlled by the anti-σ factor ChrR [22]. Orthologs of the RpoE-ChrR system are present in various bacterial species [23] including *C. crescentus* [24] *and Myxococcus xanthus* [25].

Rhizobia, soil bacteria that fix nitrogen in symbiosis with leguminous plants, are exposed to a wide range of environmental stimuli, including ROS, both in their free-living state in the soil and in the interaction with host plants, *i.e.*, during infection and establishment of symbiosis, during nitrogen fixation in root nodules, and during senescence of these nodules ( [26], ; for reviews, see [28], [29]). Accordingly, rhizobia use a set of transcription regulators to reprogram gene expression in order to cope with these stresses. Notably, during symbiosis ROS act as signaling molecules and are needed for an efficient *Rhizobium*-legume interaction [29].

**Figure 1:**
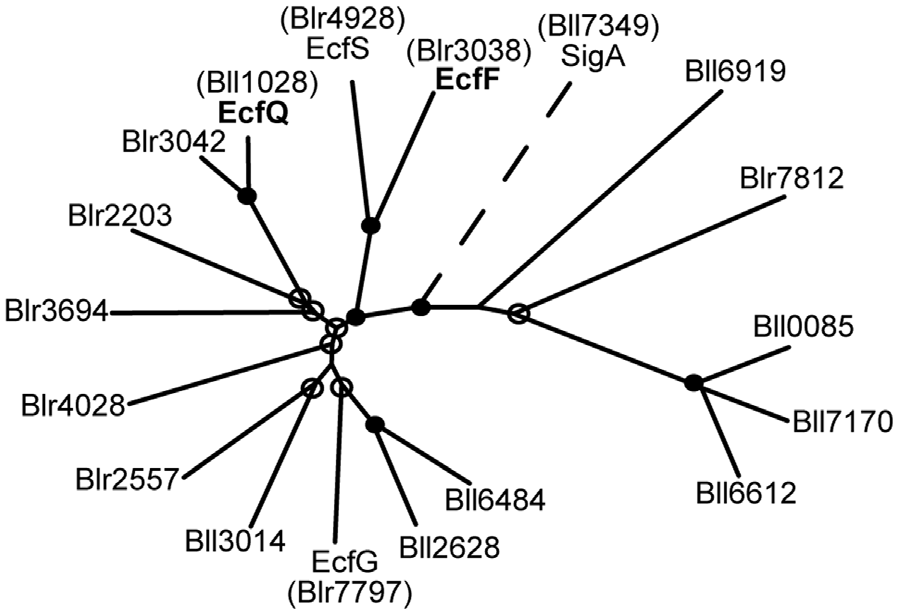
Phylogenetic relationship of 17 predicted ECF σ-factors in *B. japonicum*. The tree (generated by the UPGMA method [90]) is drawn to scale with respect to evolutionary distances. Bootstrap values were obtained after 1,000 repeats, and nodes with a confidence of greater than 90% (•) or 50% (o) are indicated. The primary σ factor (SigA) sequence of *B. japonicum* is included as an outgroup (dashed branch).

The soybean endosymbiont *Bradyrhizobium japonicum* encodes a total of 23 predicted σ factor-coding genes in its genome [30], [31]. Whereas two of them are σ^54^-type factors, 21 belong to the σ^70^ family. The latter category includes the housekeeping σ factor SigA (group 1), three RpoH σ factors (group 3), and 17 ECF σ factors (group 4) whose relationship is depicted in the phylogenetic tree shown in Figure 1. With SigA as an outgroup, the tree subdivides the ECF σ factors into two groups of 12 and 5 members documenting substantial diversity among them. In the larger group, three pairs of similar σ factors are found: EcfS (Blr4928) and Blr3038, Bll1028 and Blr3042, and Bll6484 and Bll2628, which show 35%, 55%, and 45% amino acid sequence identity, respectively. Up to now, only two members of the *B. japonicum* ECF σ family have been functionally studied in more detail. Most recently, it was described that EcfS (Blr4928) plays a critical role in the establishment of a functional symbiosis with soybean [32]. Previously, we have shown that EcfG (Blr7797) is involved in tolerance to heat and desiccation as well as in the symbiotic interaction with soybean and mungbean [31]. Other functionally studied EcfG orthologs in rhizobia include RpoE2 of *Sinorhizobium meliloti* [33] and RpoE4 of *Rhizobium etli* [34] which control regulons typical of general stress response as does σ^T^ of *C. crescentus* [35]. Similarly, ECF σ factor RpoE of the plant-associated bacterium *Azospirillum brasilense* is involved in tolerance to singlet oxygen and other abiotic stresses [36]. Yet another rhizobial ECF σ factor, RpoI of *Rhizobium leguminosarum*, is required for synthesis of the siderophore vicibactin and iron uptake [37], [38].

The transcriptome analysis of H_2_O_2_-stressed *B. japonicum* cells, which is presented here, revealed that the expression of two predicted ECF σ factors is induced by H_2_O_2_ and also in response to treatment with other ROS: Bll1028 (hereafter named EcfQ in accordance with its ECF σ factor function and annotation as CarQ in Rhizobase (http://genome-legacy.kazusa.or.jp/rhizobase/Bradyrhizobium) and Blr3038 (hereafter termed EcfF according to the SigF prototype ECF σ factor of this class [2]). We have determined the regulons of both σ factors, and demonstrated that mutant strains lacking either one or both ECF σ factor(s) show increased sensitivity to singlet oxygen. Furthermore, we have analyzed the distinct regulatory mechanisms controlling synthesis and activity of EcfQ and EcfF. While expression of both genes is controlled at the transcriptional level, activity of EcfF is additionally regulated by protein-protein interaction with its cognate anti-σ factor Blr3039 (hereafter termed OsrA for oxidative stress-response anti-σ factor). Conserved cysteine residues of OsrA are involved in H_2_O_2_ responsiveness and inhibition of EcfF activity under non-stressed conditions.

## Results

### Transcriptional Profile of B. Japonicum in Response to H_2_O_2_-mediated Oxidative Stress

In order to identify *B. japonicum* genes involved in oxidative stress response, global transcriptome analyses were performed with wild-type cells that had been treated with 2 mM H_2_O_2_ for 10 min and with untreated wild-type cells (control). To mimic the symbiotic environment we used micro-oxic conditions as standard condition for all microarray analysis throughout this study. A total of 225 genes were differentially expressed in response to H_2_O_2_ (144 upregulated, 81 downregulated; see Table S1), with 56% of them encoding proteins of unknown functions. Several genes known to be involved in the oxidative stress response were upregulated, such as catalase (blr0778), hydroperoxide resistance proteins (bll4012, bll0735) and methionine sulfoxide (MetSO) reductases (bll5855, blr7043). Notably, almost one third (29 genes) of the H_2_O_2_-regulated genes that encode proteins of known or predicted function are transcriptional regulators including five MarR-, four TetR-, and three LysR-type proteins. Furthermore, transcription of genes for three σ factors was affected by H_2_O_2_ exposure. While blr1883 encoding one of two σ^54^-type σ factors of *B. japonicum* was slightly down-regulated, the genes for two ECF σ factors *ecfQ* and *ecfF* were strongly induced (34.8 and 14.4 fold, respectively). The latter σ factors are the primary focus of this study, and some of their characteristic features are summarized in Table 1.

**Table 1:** ECF σ and anti-σ factors studied in this work.

	EcfQ	EcfF	OsrA
Locus name^a^	bll1028	blr3038	blr3039
Gene symbol^a^	*carQ*	*sigD*	–
Annotation^a^	RNA polymerase σ factor	ECF family σ factor	hypothetical protein
No. of amino acids^a^	203	186 (170^b^)	212
ECF group^c^	33	16	n.a.^d^
Distribution^c^	α-Proteobacteria	Proteobacteria	Proteobacteria
Paradigm of ECF group	–	*C. crescentus* SigF	n.a.^d^
Functional domains	σ^70^ region 2 σ^70^ region 4	σ^70^ region 2 σ^70^ region 4	DUF1109 (six transmembrane domains)
Genetically linked anti-σ factor^a^	–	OsrA	n.a.^d^

a According to [30].

b Based on the transcription start site mapped in this study, translation of *ecfF* is likely to start at a distal start codon, which leads to a shorter gene product of 170 amino acids (for details see text and Figure 6A,C).

c According to [2].

d Not applicable.

### Response of EcfQ and EcfF to Different ROS

To validate microarray data obtained for *ecfQ* and *ecfF*, and to gain insight into the expression of *ecfQ* and *ecfF* upon treatment with other sources of ROS, quantitative, cDNA-based real-time PCR (qRT-PCR) analyses were performed. Besides treatment with H_2_O_2_, the following two reagents were used: paraquat (methylviologen) generating superoxide, and rose bengal in combination with light, generating singlet oxygen (^1^O_2_). The results shown in Table 2 document induction of *ecfQ* and *ecfF* not only in response to H_2_O_2_ but also to singlet oxygen. Expression of *ecfQ*, but not *ecfF*, is also elevated under treatment with paraquat.

**Table 2:** Fold-change values of *ecfQ* and *ecfF* expression in response to different sources of ROS determined by qRT-PCR.

Experiment^a^	Treatment	Fold-change values	
		*ecfQ*	*ecfF*
1	2 mM H_2_O_2_, 10 min	104.9±30.4	18.4±4.5
2	0.2 mM paraquat, 5 min	5.9±1.1	1.5±0.2
3	0.2 mM paraquat, 10 min	8.0±1.5	0.56±0.04
4	0.5 µM rose bengal, 20,000 lux, 10 min	5.1±0.5	1.1±0.1
5	0.5 µM rose bengal, 20,000 lux, 180 min	15.4±0.7	8.0±0.6
6	Light only 20,000 lux, 60 min	1.1±0.5	1.5±0.4

a Micro-oxically grown wild-type cells exposed to H_2_O_2_ (experiment 1) or to paraquat (2, 3) for the indicated time were compared to untreated cells. Similarly, cells exposed to rose bengal plus light were compared to cells exposed to rose-bengal in the dark (4,5). In the control experiment (6), light-exposed cells were compared to cells grown in the dark. For details, see Materials and Methods.

### Phenotypic Characterization of Deletion Mutants ΔecfQ, Δ(ecfF-osrA), ΔosrA, and Δ(ecfQ, ecfF-osrA)

**Figure 2:**
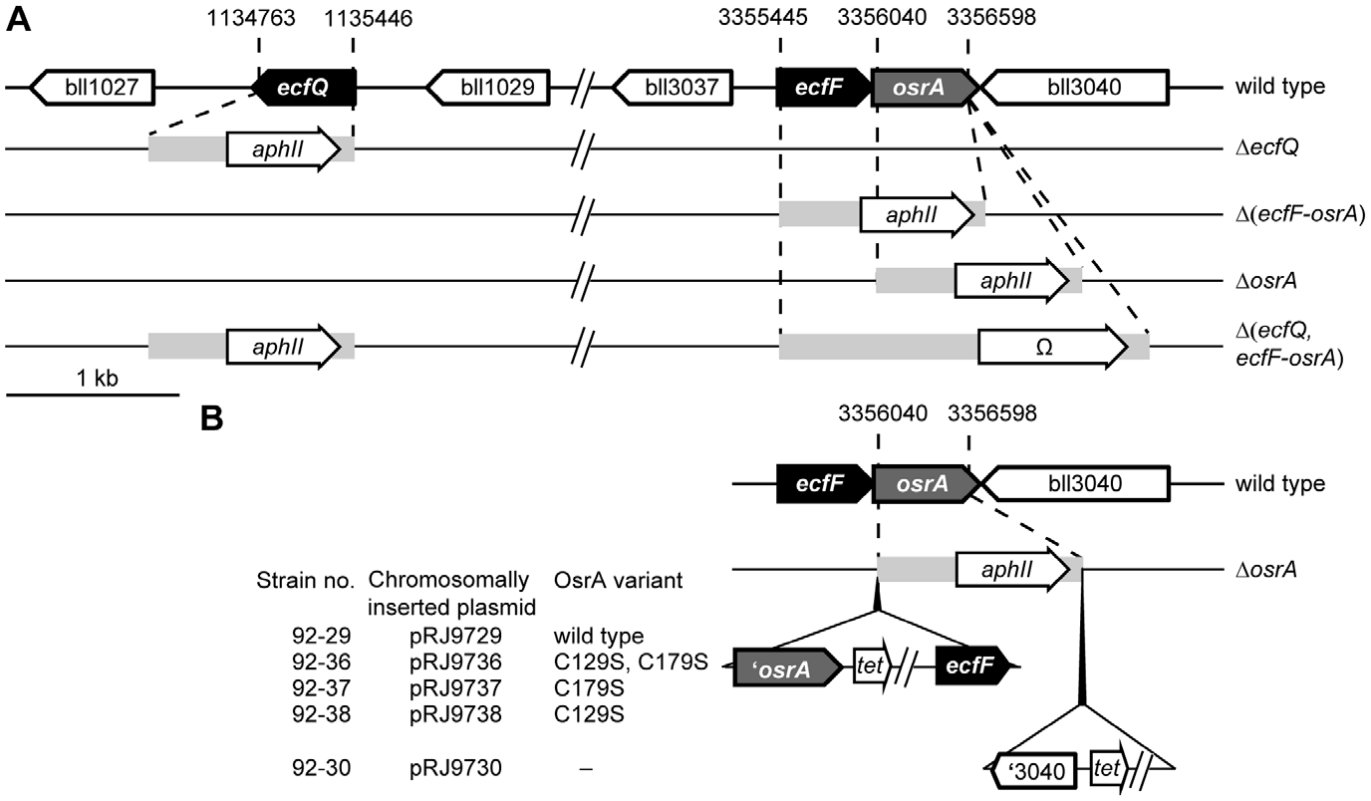
Genetic map of the *ecfQ* and *ecfF* loci in *B. japonicum* wild type and mutant strains. **A**. Genotype of deletion mutant strains. Indicated are genes coding for ECF σ factors EcfQ and EcfF (black), the putative membrane-associated anti-σ factor OsrA (grey), a predicted cytochrome *c* biogenesis protein Bll1027, a response regulator Bll1029, and for hypothetical proteins Bll3037 and Bll3040. Below the wild-type region, the genotype of mutants Δ*ecfQ* (strain 0202), Δ(*ecfF*-*osrA*) (9688), Δ*osrA* (9692) and Δ(*ecfQ*, *ecfF*-*osrA*) (15-02) is shown. In all mutants, almost the entire coding region of the deleted genes was replaced by a kanamycin (*aphII*) or spectinomycin/streptomycin (*spc/str*) resistance gene present on respective cassettes (light grey bars; for more details, see Materials and Methods). Genome coordinates refer to start and end points of deletions. **B**. Genotype of complemented derivatives of Δ*osrA* mutant. Vertical black arrowheads indicate locations where the indicated plasmids comprising a tetracycline resistance gene (*tet*) and *osrA* variants used for complementation experiments were inserted. Note that the chromosomally inserted plasmids are not drawn to scale relative to the rest of the figure.

To further elucidate the role of ECF σ factors EcfQ and EcfF in oxidative stress response, mutant strains Δ*ecfQ*, Δ(*ecfF-osrA*) and Δ(*ecfQ, ecfF-osrA*) were constructed (Figure 2). In addition, strain Δ*osrA* was generated to study the predicted function of OsrA as an anti-σ factor of EcfF. Finally, a deletion strain lacking Blr3042 was constructed to elucidate the function of this EcfQ paralog (Figure 1). As the latter strain was indistinguishable from the wild type in all phenotypic tests, it will not be further discussed in this work.

**Figure 3:**
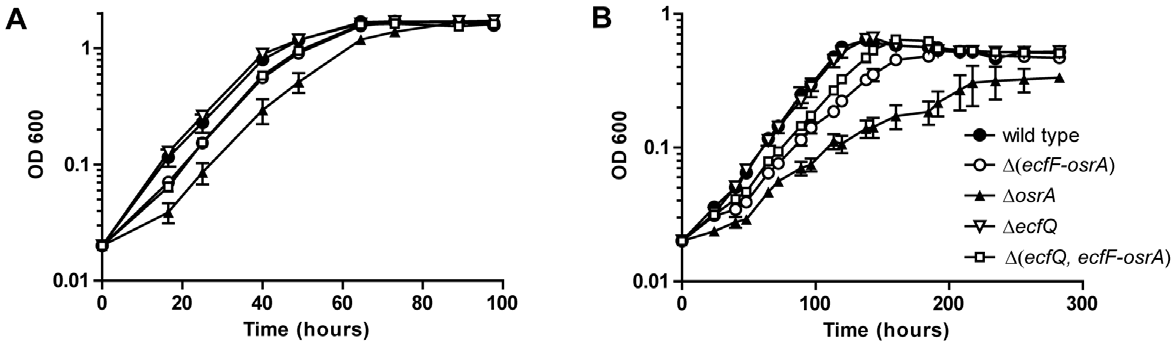
Growth characteristics of *B. japonicum* wild type and mutant strains. Bacterial cultures of *B. japonicum* wild type (•) and mutant strains Δ(*ecfF*-*osrA*) (○), Δ*osrA* (▴), Δ*ecfQ* (Δ), and Δ(*ecfQ*, *ecfF*-*osrA*) (□) were grown aerobically (**A**; PSY medium) or anaerobically (**B**; YEM medium). Data points are means of three cultures grown in parallel with bars representing standard errors of the means.

Growth kinetics of Δ*ecfQ*, Δ(*ecfF-osrA*), Δ*osrA* and Δ(*ecfQ, ecfF-osrA*) strains were determined under oxic, micro-oxic and anoxic conditions. Growth of the mutant strains followed a similar trend as seen with the wild type under micro-oxic conditions (data not shown). In oxic and anoxic conditions, growth of strains Δ(*ecfF-osrA*), Δ*osrA* and Δ(*ecfQ, ecfF-osrA*) but not of Δ*ecfQ* was retarded compared to the wild type (Figure 3A,B). In anoxic conditions doubling time of strain Δ*osrA* was approximately twice that of the wild type, and the final optical density reached by this mutant was lower (Figure 3B).

All mutant strains were symbiotically proficient and indistinguishable from the wild type when tested on two different soybean varieties (*Glycine max* cultivar Williams 82 and cultivar „Green Butterbean“), on mungbean (*Vigna radiata*) and on cowpea (*Vigna unguiculata*) (data not shown).

**Figure 4:**
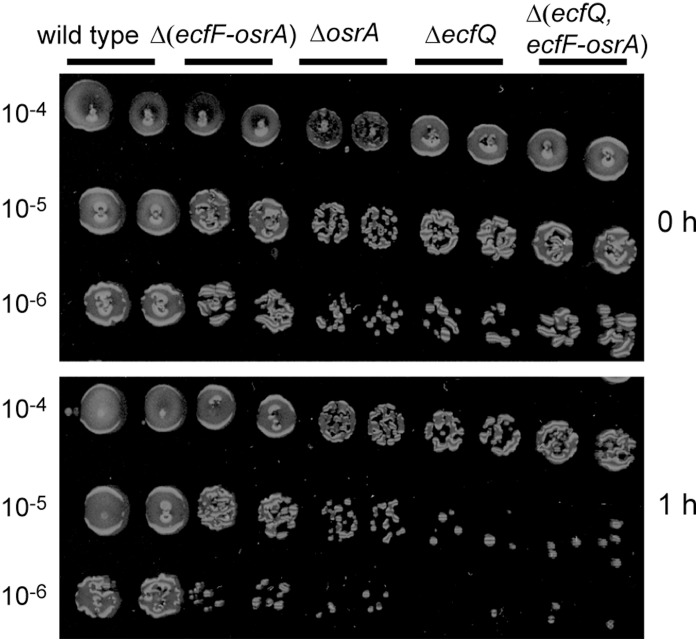
Singlet oxygen sensitivity test. Cultures of *B. japonicum* wild type and mutant strains Δ*ecfQ*, Δ(*ecfF*-*osrA*), Δ*osrA* and Δ(*ecfQ*, *ecfF*-*osrA*) were pre-grown to early stationary phase, and aliquots of serial dilutions were spotted on plates containing 0.1 µM rose bengal (two independent dilution series per strain). The control plate shown in the upper panel was incubated in the dark while the plate shown in the lower panel was light exposed (2,000 lux) for 1 h to allow generation of singlet oxygen (for more details, see Materials and Methods).

Further, the sensitivity of the mutants towards different ROS was tested in filter disk assays, on gradient plates and in spot tests. All four mutant strains showed increased sensitivity towards oxidative stress caused by singlet oxygen both on gradient plates (data not shown) and when spotted on PSY plates containing rose bengal (Figure 4). In filter disk assays, the mutants showed a wild-type phenotype with respect to their sensitivity to the following compounds: (i) H_2_O_2_; (ii) diamide, a reactive electrophilic species which affects the thiol redox balance; (iii) FeSO_4_ that can generate oxidative stress via the Fenton reaction; (iv) NO-generating agents such as *S*-nitroso-*N*-acetylpenicillamine and *S*-nitrosoglutathione; (v) methylglyoxal, a toxic, electrophilic compound; (vi) paraquat (methylviologen) which causes the formation of superoxide (data not shown).

### The Regulon of EcfQ

Microarray analysis was used to identify potential target genes of EcfQ. To this end RNA was isolated from the wild type and the Δ*ecfQ* strain, both grown unstressed or stressed by exposure to H_2_O_2_. Expression of nine genes differed between the wild type and the Δ*ecfQ* mutant under non-stressed conditions (four up-regulated, five down-regulated; Table S2A). In H_2_O_2_-stressed cells, the number of differentially expressed genes increased to 34 with seven genes up-regulated and 27 genes down-regulated (Table S2B). The latter category might include direct targets of EcfQ given the positive regulation mode exerted by σ factors. However, inspection of the DNA regions (200 bp) upstream of these genes did not reveal common motifs that might function as recognition site of EcfQ. Notably, two thirds of the differentially regulated genes encode hypothetical or functionally unknown proteins. Among genes with predicted functions are blr0337 and blr3534 which code for a subunit of putative carbon monoxide dehydrogenases and are both down-regulated in the mutant.

### The Promoter Region of ecfQ and of other Genes Coding for Class 33 ECF σ Factors are Conserved

**Figure 5:**
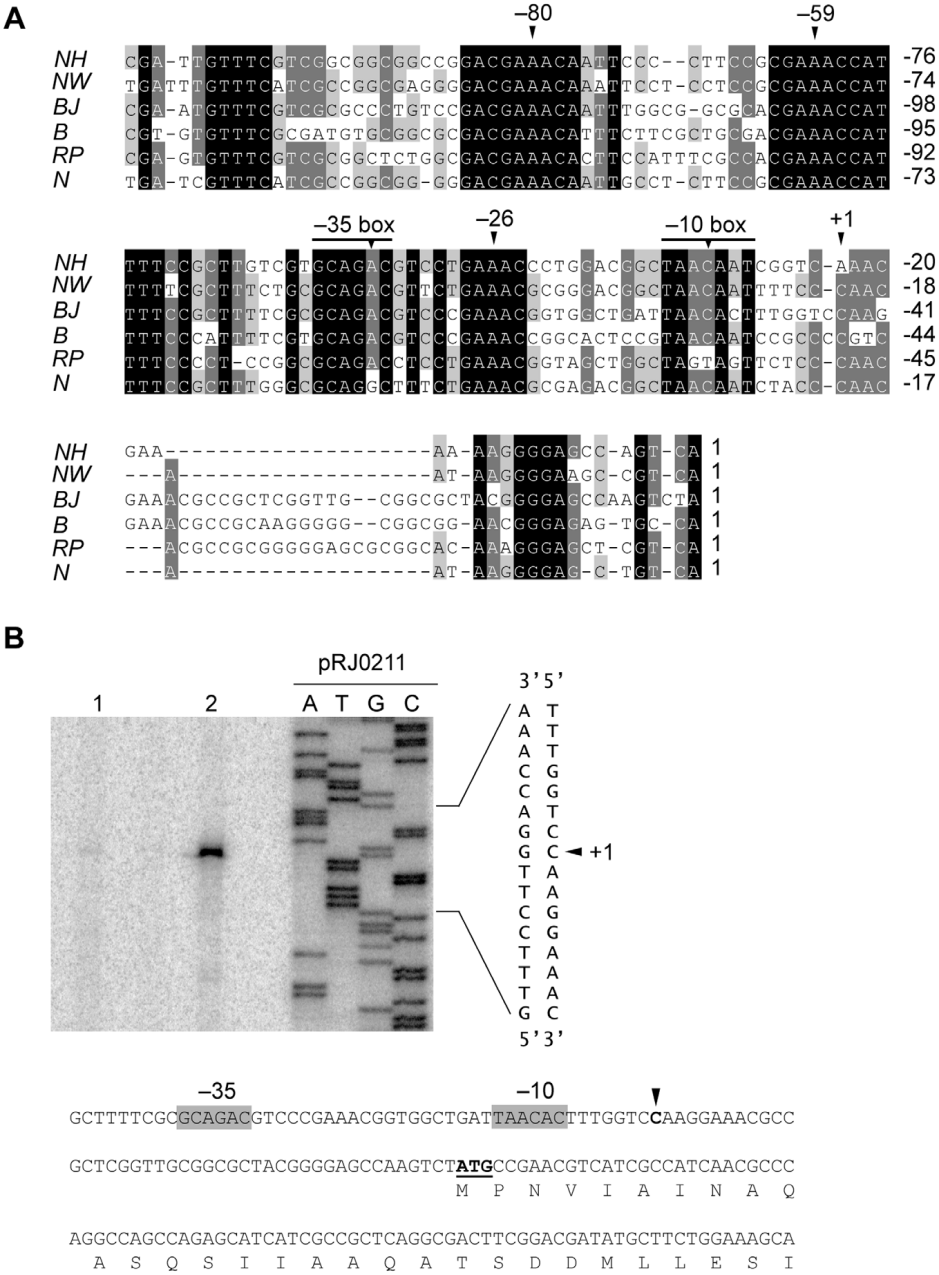
Analysis of the *ecfQ* promoter region. **A**. Alignment of the upstream regions of six α-proteobacterial genes coding for class 33 ECF σ factors, which lack associated anti-σ factor genes. Numbers on the right of each line refer to the position of the last nucleotide in the line within the corresponding upstream region to the annotated translational start sites. The transcription start site of *ecfQ* is labeled “+1″. Shaded in black, dark grey, and light grey are nucleotides which are identical in all, 80%, and 60% of the sequences, respectively. Marked are the putative core promoter regions (–35, –10) and several conserved motifs (boxes –26, –59, and –80; for details, see text). GI numbers of the proteins encoded by the adjacent genes are as follows: *Nitrobacter hamburgensis* X14 (*NH*), 92119140; *Nitrobacter winogradskyi* Nb-255 (*NW*), 75677236; *Bradyrhizobium japonicum* USDA 110 (*BJ*), 27376139; *Bradyrhizobium sp.* BTAi1 (*B*), 148252297; *Rhodopseudomonas palustris* CGA009 (*RP*), 39937850; and *Nitrobacter sp.* Nb-311A (*N*), 85713893. **B**. 5′ end mapping of *ecfQ* mRNA. For primer extension total RNA of the wild-type strain grown under the following conditions was used: micro-oxic (lane 1), and micro-oxic, treated with 2 mM H_2_O_2_ for 10 min (lane 2). Extension products obtained with the [^32^P]-labeled primers pe-1028-1 and pe-1028-2 were separated on a 6% denaturing polyacrylamide gel (only results obtained with primer pe-1028-1 are shown). The sequencing ladder was generated with plasmid pRJ0211 and primer pe-1028-1 (for more details, see Materials and Methods and Table S6). Part of the predicted promoter region is shown on the right, with the experimentally assigned transcription start site indicated with an arrowhead. The sequence of the *ecfQ* promoter region is shown below the picture. The determined 5′ end of the transcript is indicated by an arrowhead, and the annotated translation start site is underlined and printed in bold face. Putative −10 and −35 regions are shaded in grey. The N-terminus of the EcfQ protein sequence is indicated in one-letter code.

When we had a closer look at the 13 α-proteobacterial genes representing the class 33 of ECF σ factors to which EcfQ belongs [2] we made several observations: (i) in almost every organism of this group, except *Mesorhizobium loti*, there are two genes coding for this type of ECF σ factor; (ii) consistently, one of them has a predicted anti-σ factor gene in its proximity, but for the other, a predicted anti-σ factor gene is absent (EcfQ together with five other class 33 σ factors belongs to the latter category); (iii) by aligning the upstream regions of the six genes of this second group, a striking pattern of sequence conservation was observed (Figure 5A). To obtain information on the relative position of these elements in the promoter, the 5′ end of *ecfQ* mRNA was determined by primer extension, using RNA isolated from the *B. japonicum* wild type grown under different conditions (Figure 5B). The results of reverse transcription revealed the *ecfQ* transcription start point at a C located 44 nucleotides upstream of the annotated *ecfQ* start codon (Figure 5B). In agreement with the microarray and qRT-PCR analyses, the amount of cDNA derived from RNA in H_2_O_2_-treated cells (lane 2) was higher than the amount derived from untreated cells (lane 1). Putative −35 and −10 promoter boxes were identified, forming the consensus GCAGAC and TAACAAT, respectively, however, the spacing between the motifs is unusually long (20 nt).

Several additional stretches of nucleotides are also conserved in the upstream region of *ecfQ* and the other five σ factor-coding genes which belong to the same group. A stretch reading GAAAC is repeated several times in the upstream region (boxes labeled −26, −59, and −80 in Figure 5A). At box −80, the GAAAC sequence is part of the inverted repeat TGTTTC-N_17_−GAAACA (Figure 5A). Database searches with the virtual footprint tool Prodoric to find regulators that might bind to this region revealed no obvious candidates. Also, the identified region does not resemble any described binding sites for several *B. japonicum* regulators such as Irr, Fur, FixK_2_, and RegR [39]–[41].

### The Regulon of EcfF

To identify genes possibly controlled by σ factor EcfF, microarray analyses were performed with the Δ(*ecfF-osrA*) mutant strain. In micro-oxically grown, unstressed mutant cells, expression of only three genes was slightly up-regulated apart from the obvious decrease of expression of the two deleted genes (Table S3A). This indicated that in the wild type EcfF is mainly inactive under these growth conditions. Upon H_2_O_2_ treatment, expression of 22 genes (including *ecfF* and *osrA*) differed between mutant and wild-type cells confirming that EcfF-dependent transcription is activated by H_2_O_2_ exposure (Table S3B). Notably, all regulated genes had negative fold-change values, which is in line with the role of EcfF as a positive regulator and suggests that there are direct target genes in this group. Other than *ecfF* and *osrA* no genes were common to the lists of differentially expressed genes in unstressed and stressed cells.

To examine the predicted function of OsrA as an anti-σ factor, we also performed microarray experiments with the Δ*osrA* mutant strain. We used unstressed, micro-oxically grown cells in these experiments because we assumed that even in unstressed cells the absence of the anti-σ factor OsrA should result in up-regulation of EcfF-dependent genes if the function of OsrA in the wild type were to inhibit the activity of EcfF under these conditions. Expression of 39 *B. japonicum* genes (including *ecfF* and *osrA*) was altered in the Δ*osrA* strain, with 24 genes (more than 60%) encoding hypothetical or unknown proteins (Table S4). Only 3 genes had negative fold-change values (one of them being the deleted *osrA* gene) while the large majority of 36 genes was up-regulated, likely due to hyperactivity of EcfF caused by the absence of its anti-σ factor. Elevated expression of *ecfF* (18.8 fold) in the Δ*osrA* background suggested that the *ecfF*-*osrA* operon is autoregulated. Interestingly, not only *ecfF* but also its paralog *ecfS* (blr4928) was more highly expressed (3.8 fold) in the mutant pointing to potential cross-talk between the two σ factor−anti-σ factor systems.

When the expression data generated with stressed cells of the Δ(*ecfF-osrA*) mutant was compared with that of unstressed Δ*osrA* cells, nine genes (apart from the mutated genes) showed a regulatory pattern that is expected for EcfF-OsrA being a cognate σ factor−anti-σ factor pair, *i.e.*, down-regulation in the Δ(*ecfF-osrA*) mutant and up-regulation in the Δ*osrA* mutant (Table 3). Except for blr7044, all genes of this group belong to the H_2_O_2_-inducible genes. Notably, genes bll5855, blr7043, blr7044 all encode predicted peptide MetSO reductases.

**Table 3:** *B. japonicum* genes whose expression differed in the Δ(*ecfF*-*osrA*) and the Δ*osrA* relative to the wild type^a^.

Gene no.^b^	Fold change			Known or predicted gene product^d^
	wt^c^	Δ(*ecfF*-*osrA*)	Δ*osrA*	
*bll1027*	7.8	−15.4	89.5	putative cytochrome *c* biogenesis protein
* bll1026*	11.4	−20.1	92.4	hypothetical protein
bsr4431	10.7	−15.9	20.0	hypothetical protein
bll5855	7.5	−6.2	20.6	peptide methionine sulfoxide reductase
bll6527	4.7	−4.7	78.3	hypothetical protein
*blr7043*	8.5	−7.0	13.5	peptide methionine sulfoxide reductase
* blr7044*	−	−2.9	5.4	peptide methionine sulfoxide reductase
* bsr7045*	3.2	−2.9	4.1	hypothetical protein
blr7741	19.3	−28.3	54.2	hypothetical protein
*ecfF*	14.4	−60.6	18.8	σ factor EcfF
* osrA*	8.1	−210.1	−19.9	anti-σ factor OsrA

a Cells were grown micro-oxically and those of strain Δ(*ecfF*-*osrA*) were exposed to 2 mM H_2_O_2_ for 10 min prior to harvest. Wild-type cells grown under the respective conditions served as reference in both experiments. Listed are genes with an absolute fold-change value of >3 in at least one of the mutants and >2 in the other mutant.

b Nomenclature according to [30]. Putative operons are shown in italics with co-transcribed promoter-distal genes indented to the right.

c Fold-change values from the comparison of micro-oxically grown wild-type cells exposed to 2 mM H_2_O_2_ for 10 min with untreated cells (see Table S1).

d Gene description according to [30] with modifications.

**Figure 6:**
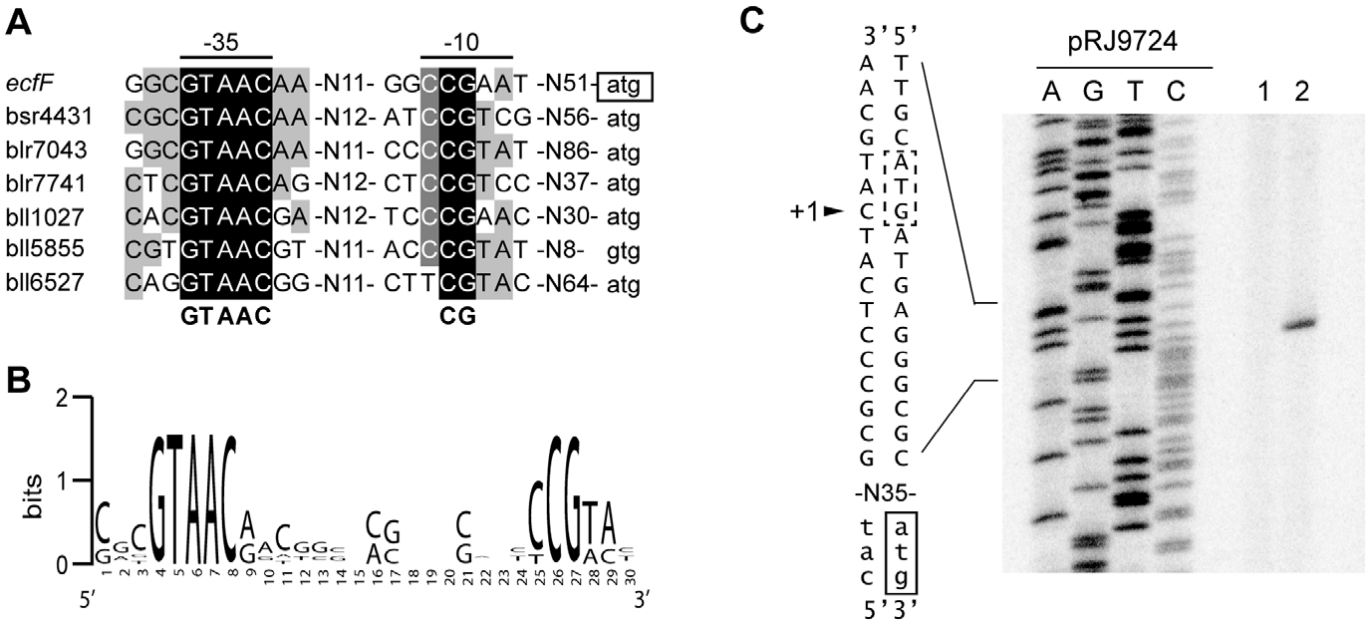
*B. japonicum* EcfF-target promoter motif located in the upstream regions of OsrA-regulated transcription units. **A**. Alignment of nucleotide sequences located upstream of seven EcfF-regulated transcription units. Names of promoter-proximal genes are indicated on the left. Nucleotides shaded in black, dark grey or light grey are conserved in all, in six, or at least in four of the sequences, respectively. Most conserved nucleotides are also shown in bold under the alignment. Annotated start codons are shown in lower-case with that of *ecfF* (boxed) being reannotated based on the result of the transcript mapping data shown in Figure 6C (for details see text). **B**. WebLogo of the EcfF-target promoter base on the alignment from panel A. **C**. 5′ end mapping of *ecfF*. For primer extension, total RNA of the wild-type strain grown under the following conditions was used: micro-oxic (lane 1), and micro-oxic, treated with 2 mM H_2_O_2_ for 10 min (lane 2). Extension products obtained with the [^32^P]-labeled primers pe-3038-1 and 3038-RT-R were separated on a 6% denaturing polyacrylamide gel (only results obtained with primer pe-3038–1 are shown). The sequencing ladder was generated with plasmid pRJ9724 and primer pe-3038–1 (for details, see Materials and Methods and Table S6). Part of the predicted promoter region is shown on the left, with the experimentally assigned transcription start site “+1″ indicated with an arrowhead. Because the ATG translation start codon of *ecfF* as annotated in Rhizobase ( [30]; dashed-line rectangle) overlaps the transcription start, the more distal, alternative start codon seems more likely (solid-line rectangle).

Taking into account the predicted operon structure for bll1027-26, *ecfF-osrA* and blr7043-45, the genes listed in Table 3 comprise a total of seven transcription units with promoters that are primary candidates for being direct EcfF targets. When we searched in a 200-bp window upstream of the respective start codons for common putative promoter elements we could indeed identify a conserved GTAAC(g,a)–N_14-15_–(c,t)CG(t,a) motif (Figure 6A,B). This element is remarkably similar to the tGTAACc–N_16_–CGAA promoter sequence that was proposed for group 16 of ECF σ factors [2] to which EcfF belongs. The predicted EcfF target promoter preceding *ecfF-osrA* was confirmed by primer extension experiments (Figure 6C). Indeed, a transcript starting at a C located 6 bp downstream of the predicted –10 box of *ecfF* was detected in cells exposed to H_2_O_2_ but not in untreated cells. The experimentally detected transcription start site overlaps the *ecfF* ATG start codon annotated in Rhizobase [30] which argues for the more distal translational start codon as indicated in Figure 6C. Likewise, the annotated GTG start codon of bll5855 might be incorrect because the predicted −10 box of the respective promoter is located only 8 bp upstream of this start codon (Figure 6A). When the EcfF consensus motif (GTAAC(g,a)–N_14–15_–(t,c)CG(t,a); Fig. 6B) was used as a query for a genome-wide *in silico* search (for details, see Materials and Methods) a total of 18 hits were identified of which 7 are associated with the genes or operons listed in Table 3. The remaining 11 motifs precede genes that did not fulfill the selection criteria applied to the genes included in Table 3. Those 11 hits either represent false positives, or EcfF-mediated regulation of the associated genes is masked by other unknown regulatory effects.

### In Vivo Interaction of EcfF and OsrA

**Figure 7:**
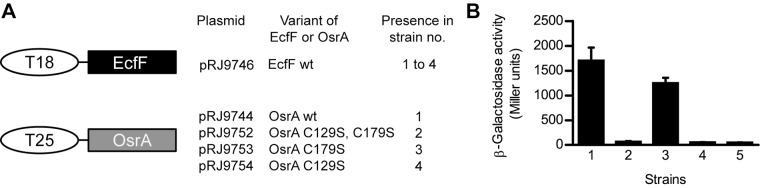
Interaction between EcfF and OsrA monitored in a bacterial two-hybrid system. **A**. Schematic representation of analyzed hybrid proteins. *B. pertussis* adenylate cyclase fragments T18 and T25 (oval shaped) were translationaly fused to σ factor EcfF (black rectangle) and anti-σ factor OsrA (grey rectangle; wild-type (wt) or mutant variants), respectively. Plasmids encoding respective proteins were transformed into *E. coli* BTH101 in the indicated combinations to yield strains 1 to 4. **B**. *E. coli* cultures were grown for 18 h at 30°C and assayed for β-galactosidase activity. Control strain 5 containing vectors pKT25 and pUT18C was used to determine background activity. Shown are mean values and standard deviations derived from a representative experiment with four independent cultures per strain.

If EcfF and OsrA functioned as a typical cognate σ factor–anti-σ factor pair they ought to interact directly at the protein level. We have used a bacterial two-hybrid system (BACTH system; [42], [43]) to further evaluate this model. Plasmids pRJ9746 and pRJ9744 encoding protein fusions of EcfF and OsrA to adenylate cyclase Cya subdomains T18 and T25, respectively, were constructed (Figure 7A). Cotransformation of *E. coli* BTH101 cells with these plasmids resulted in strain 1 which showed significant β-galactosidase activity (Figure 7B). By contrast, no β-galactosidase activity above background was detected in *E. coli* BTH101 cells that contained either of the fusion plasmid in combination with the empty vector of the other hybrid plasmid (data not shown). This indicated that interaction of EcfF with OsrA enabled functional complementation of the T18 and T25 adenylate cyclase domains.

### Conserved Cysteine 129 of OsrA Might be Required for Interaction with EcfF

**Figure 8:**
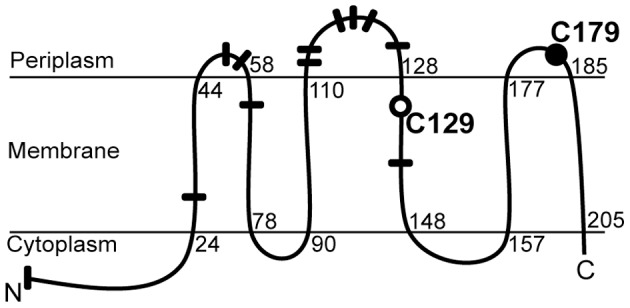
Topology model of OsrA. The figure shows the predicted topology of anti-σ factor OsrA and localization of cysteine residues 129 and 179 which are highly conserved among anti-σ factors associated with class 16 ECF σ factors. Numbers refer to amino acid positions at the beginning and the end of six transmembrane-spanning domains. The OsrA portion from amino acid 10 to 212 is annotated as DUF1109 (domain of unknown function). Results presented in this work suggest that Cys-129 (open circle) is needed for OsrA to interact with EcfF while Cys-179 (solid circle) is required for the response to hydrogen peroxide. Black bars mark twelve methionine residues of which eight are predicted to map to periplasmic loops.

Amino acid sequence alignment of OsrA with orthologous putative anti-σ factors associated with group-16 σ factors in other proteobacteria revealed two highly conserved cysteine residues. These residues are located at positions 129 and 179 of OsrA, and they are the only cysteines present in this protein (Figure 8, Figure S1).

To probe the function(s) of the conserved cysteines, mutant variants of OsrA (C129S, C179S, C129S+C179S) were fused to T25 (Figure 7A) and tested for two-hybrid interaction in combination with the T18-EcfF fusion protein. In strain 3 harboring the T25-OsrA C179S fusion, β-galactosidase activity reached about 70% of the reference strain 1 (T25-OsrA) whereas in strains 2 (T25-OsrA C129S, C179S) and 4 (T25-OsrA C129S) only background activity was detected (Figure 7B). Assuming that the point mutations did not drastically alter protein expression levels or stability, these results imply that cysteine 129 of OsrA, but not cysteine 179, is required for interaction with EcfF.

### Cysteine 179 of OsrA is Required for the H_2_O_2_ Response of EcfF in B. Japonicum

To validate the data obtained with the *E. coli*-based two-hybrid system in *B. japonicum* and for further functional analysis of the conserved cysteines of OsrA, expression of the autoregulated *ecfF* gene was monitored in derivatives of the Δ*osrA* strain complemented with wild-type or mutant variants of OsrA. To this end, single copies of wild-type *osrA* and mutant variants (present on pSUP202pol4-based plasmids) were chromosomally integrated into the Δ*osrA* strain. The resulting strains (Δ*osrA* complemented with wild-type OsrA, OsrA C129S+C179S, OsrA C179S, or OsrA C129S) and the control strain Δ*osrA* containing the pSUP202pol4 vector integrated in the chromosome (Figure 2B), were grown under micro-oxic conditions without or with stress exposure (2 mM H_2_O_2_ 10 min) prior to cell harvest. RNA was isolated and reverse-transcribed into cDNA which was used for quantitative real-time PCR. From the results shown in Table 4 we conclude that (i) the complementation strategy is effective because wild-type OsrA restores the normal *ecfF* expression pattern (cf. Table 2); (ii) OsrA C129S and OsrA C129S+C179S are not functional because the respective strains showed a very similar *ecfF* expression pattern as the control strain which lacks OsrA; (iii) C179 of OsrA is crucial for H_2_O_2_ responsiveness because *ecfF* expression in the strains complemented with OsrA C179S or wild-type OsrA was very similar under non-stressed conditions; yet in the former strain, no induction occurred after H_2_O_2_ exposure.

**Table 4:** Regulation of *ecfF* in a *B. japonicum* Δ*osrA* background complemented with wild-type or mutant versions of *osrA*.

Strain	OsrA variant tested for complementation of Δ*osrA*	Fold-change values^a^	
		No stress	H_2_O_2_-stress
92–30^b^	–	16.3±3.4	11.6±2.0
92–29	wild-type	1	15.3±3.0
92–38	C129S	19.8±6.4	20.5±6.1
92–37	C179S	1.2±0.3	1.1±0.3
92–36	C129S, C179S	14.8±5.6	22.1±6.7

a Cells were grown micro-oxically without stress or were exposed to 2 mM H_2_O_2_ for 10 min prior to harvest. Expression levels of *ecfF* in different backgrounds were determined by qRT-PCR and expressed as fold-change values ± standard errors relative to the expression detected in the pseudo wild-type strain 92-29 under non-stress conditions. Data are based on three technical replicates of a representative experiment which was repeated in three biological replicates. For details, see Materials and Methods.

b Control strain containing vector pSUP202pol4 chromosomally integrated downstream of the Δ*osrA::aphII* locus (see Figure 2B).

## Discussion

Rhizobia are exposed to oxidative stress originating from ROS that are generated either intrinsically in aerobic metabolism or by legume host plants during rhizobial infection [29]. Here we have analyzed the transcriptional response of *B. japonicum* cells to oxidative stress. Special emphasis was given to the two ECF σ factors EcfQ and EcfF because (i) their transcription was strongly induced upon exposure to ROS, and (ii) ECF σ factors are typical regulators in the bacterial stress response. Hydrogen peroxide exposure of free-living cells which were grown micro-oxically to mimic symbiotic conditions resulted in altered transcription of more than 200 genes. Many of them are functionally uncharacterized, others are related to oxidative stress or encode transcriptional regulators. Among the latter category are five MarR-type and three LysR-type regulators which are involved in the oxidative stress response in various other bacteria [44]–[48].

In recent studies, effects of H_2_O_2_ and paraquat exposure on transcription in oxically grown *B. japonicum* cells were described [49], [50]. A comparison of that study with our own results revealed that genes induced by H_2_O_2_ in both oxic and micro-oxic cells comprise those encoding hydroperoxide resistance proteins (bll4012, bll0735), putative epoxide hydrolase 1 (bll3418), a putative glutathione S-transferase (bll7849), and the ECF σ factors mentioned above (e*cfQ*, *ecfF*). Fold-change values differed substantially between the two studies which is likely due to different growth conditions and different microarray platforms. Remarkably, the gene for catalase KatG (blr0778), whose role in protection from oxidative stress in *B. japonicum* is well documented [51], appeared to be induced by hydrogen peroxide treatment only in micro-oxically but not in oxically grown cells. We speculate that blr0778 is induced even in untreated aerobic cells due to endogenous ROS production that might be higher in oxic cells than in micro-oxic cells.

ECF σ factors EcfQ and EcfF were functionally characterized by phenotypic analysis of respective mutants and microarray analyses. Deletion mutants Δ*ecfQ*, Δ(*ecfF*-*osrA*), Δ*osrA* and Δ(*ecfQ*, *ecfF*-*osrA*) were more sensitive to singlet oxygen, and thus confirmed that both σ factors are indeed involved in oxidative-stress tolerance. Singlet oxygen sensitivity of the mutants was moderately increased and restricted to this type of ROS. This might be due to an intrinsic tolerance of *B. japonicum* and/or the existence of functionally redundant, EcfQ−/EcfF-independent ROS-protective systems. This hypothesis is in line with the finding that the regulons of EcfQ and EcfF showed only limited overlap with the large group of H_2_O_2_-responsive genes (Figure S2), and it also could explain the symbiotic proficiency of the mutants.

In the absence of stress, both σ factors are probably inactive because under these conditions regulation of only few genes was altered in the deletion mutants, possibly by indirect means as none of them was differentially expressed in stressed cells (Tables S2 and S3). Nevertheless, growth of the mutant lacking OsrA was impaired even without externally applied oxidative stress, particularly under anoxic conditions, which indicates that hyperactivity of EcfF might be deleterious.

Transcriptional control of *ecfQ* and *ecfF* is likely to occur via different mechanisms. The presence of conserved motifs within the *ecfQ* promoter region points to the involvement of a yet unidentified transcriptional regulator, a model that is compatible with the absence of an anti-σ factor gene associated with *ecfQ*. By contrast, the *ecfF*-*osrA* operon appears to be autoregulated, which is typical for cognate σ and anti-σ factors genes organized in an operon. The difference in the regulatory mode may also be responsible for the differential response of these σ factor genes to treatment with paraquat.

EcfQ and EcfF control rather small and largely distinct groups of genes. Common to both regulons are only four clustered genes (bll0331–0333; blr0337) of which bll0333 encodes a precursor of a putative alcohol dehydrogenase and blr0337 a subunit of a predicted carbon monoxide dehydrogenase. Notably, genes blr0335 and blr0336 encoding two additional subunits of the latter enzyme are also controlled by EcfF. The regulon of EcfQ is functionally rather undefined because almost 70% of its members are hypothetical or unknown proteins. By contrast, more than 70% of the proteins encoded by the genes belonging to the EcfF regulon have a (predicted) functional annotation. Strikingly, half of them are oxidoreductases including MetSO reductases Bll5855 and Blr7043 whose genes were induced by H_2_O_2_ treatment. A third MetSO reductase, Blr7044, that is induced 2.9-fold by H_2_O_2_ exposure, is yet another member of the EcfF regulon as its expression was inversely affected in the Δ(*ecfF*-*osrA*) and Δ*osrA* strains (Table 3). Thus, at least three of five MetSO reductases encoded in the *B. japonicum* genome are H_2_O_2_ responsive and controlled by EcfF/OsrA (Blr0834 and Bll6260 being the remaining two). *Neisseria gonorrhoeae* [52] and *Neisseria meningitidis* [53] represent two other bacterial species where genes encoding MetSO reductases are controlled by an ECF σ factor–anti-σ factor pair.

For many bacterial species the function of MetSO reductases as antioxidant repair enzyme is well documented [54]–[59]; for review, see [60]). Repair of oxidized methionines by MetSO reductases depends on protein electron donors such a thioredoxin (for review, see [61]). Based on its putative signal sequence Blr7043 is predicted to localize to the periplasm. Thus, for Blr7043 to function additional components are required which transfer electrons across the cytoplasmic membrane and deliver them to this enzyme. DsbDC of *E. coli* is a well characterized example which is needed for reduction or isomerization of disulfide bonds in the periplasm [62]. Based on the predicted topology and domain structure of Bll1026 (membrane-anchored periplasmic thioredoxin) and Bll1027 (membrane protein with a DsbD β core domain), we speculate that Blr7043 in *B. japonicum* may receive electrons via these proteins whose genes are co-regulated with blr7043 by EcfF/OsrA.

*In vivo* interaction of EcfF with OsrA was demonstrated with a bacterial two-hybrid system in *E. coli*. These experiments revealed that a conserved cysteine at position 129 of the anti-σ factor OsrA is crucial for interaction with EcfF, and this result was further substantiated by the finding that replacement of this residue led to constitutive EcfF activity in *B. japonicum*. The predicted localization of C129 in the cytoplasmic membrane argues against this amino acid making direct contact with EcfF. However, it is possible that C129 is crucial for keeping OsrA in an interaction-competent conformation and that its replacement with serine interfered with this function.

The second conserved cysteine of OsrA, C179, is probably involved in sensing and/or transducing the stress signal because inhibition of EcfF by the OsrA C179S variant was not released when *B. japonicum* cells were stressed with hydrogen peroxide. Taking into account our data and the predicted OsrA topology (Figure 8) we propose that oxidative stress detected via periplasm-exposed C179 is signaled to the cytoplasmic portion of OsrA where it leads to the release of bound EcfF. Although cysteines are redox-active amino acids and thus well suited to monitor oxidative stress, C179 of OsrA is not necessarily the primary signal input site. Inspection of the OsrA amino acid sequence revealed a striking accumulation of eight methionine residues in two predicted periplasmic loops (Figure 8), with only two of them being conserved in the closest *B. japonicum* paralog TmrS (Blr4929), the anti-σ factor of EcfS (Blr4928) (Figure 1, Figure S1; [32]). Given that three MetSO reductases are controlled by EcfF/OsrA it is tempting to speculate that the presence of multiple methionines in OsrA makes this regulator intrinsically responsive to molecules that elicit methionine oxidation.

Amino acids that are critical for oxidative stress signaling were identified previously in other anti-σ factors which, however, are not homologs of OsrA. Specifically, conserved histidine or cysteine residues in the ChrR anti-σ factor proteins of *C. crescentus*, *R. sphaeroides,* and *N. meningitidis* are required for proper regulation of the respective σ^E^ proteins in response to organic hydroperoxide [24], [53], [63]. Likewise, in *M. xanthus* blue-light responsiveness of the membrane-bound CarQ anti-σ factor is controlled by another membrane-associated protein, CarF, whose anti-anti-σ factor activity depends on several histidine residues [64], [65].

Our study contributes to the characterization of an ECF σ factor family found in *B. japonicum*. With EcfQ and EcfF studied here and the previously described σ factors EcfG [31] and EcfS [32], functional information is now available for a total of four ECF σ factors of this bacterium. While the general stress response regulator EcfG contributes to both free-living and symbiotic traits, functions of EcfS are largely confined to symbiosis and those of EcfQ and EcfF to the free-living state. The function of the EcfQ paralog Blr3042 remains enigmatic as it turned out to be dispensable under all tested conditions. Challenging goals for future studies include the characterization of signals, mechanisms of their transduction, and functions of target genes of EcfQ and EcfF.

## Materials and Methods

### Bacterial Strains and Growth Conditions

Bacterial strains used in this work are listed in Table S5. *Escherichia coli* strains were grown in Luria-Bertani medium at 37°C [66] containing these concentrations of antibiotics for plasmid selection (µg ml^−1^): ampicillin, 200; kanamycin, 30; tetracycline, 10. *B. japonicum* strains were cultivated at 30°C aerobically (21% O_2_ in the gas phase) or micro-oxically (0.5% O_2_) in peptone-salts-yeast extract (PSY) medium supplemented with 0.1% arabinose [40], or anaerobically (100% N_2_) in yeast extract-mannitol (YEM) medium containing 10 mM KNO_3_ [67], [68]. Where appropriate, antibiotics were used at these concentrations (µg ml^−1^): spectinomycin, 100; kanamycin, 100; streptomycin, 100 (solid media) and 50 (liquid media); tetracycline, 50 (solid media) and 25 (liquid media). Aerobic cultures were grown in vigorously shaken (160 rpm) Erlenmeyer flasks containing one-fifth of their total volume of PSY medium. In oxidative stress experiments, cells were exposed to 2 mM H_2_O_2_ for 10 min, conditions that do not inhibit growth as shown previously [69].

### Mutant Construction

Mutant strains 0202 (Δ*ecfQ*), 9688 (Δ[*ecfF*-*osrA*]) and 9692 (Δ*osrA*) were constructed by marker-exchange mutagenesis. Briefly, the 5′- and 3′-flanking regions of the genes to be deleted were amplified by PCR using primer pairs listed in Table S6, cloned in the pGEM-T Easy vector (Promega Corp., Madison, WI, USA), verified by sequencing, and finally cloned in tandem in vector pSUP202pol4. A 1.2-kb kanamycin resistance cassette (*aphII*) derived from pBSL86 [70] was inserted between the up- and downstream regions to generate plasmids pRJ0202 (for deletion of *ecfQ*), pRJ9688 (for deletion of *ecfF* plus *osrA*), and pRJ9692 (for deletion of *osrA*). The resulting plasmids were transformed into *E. coli* S17-1 and then mobilized by conjugation into *B. japonicum* wild-type strain 110*spc*4 as previously described [71]. The correct genomic structure of the resulting deletion mutants 0202 (Δ*ecfQ*), 9688 (Δ[*ecfF*-*osrA*]), and 9692 (Δ*osrA*) was verified by PCR. In strains 9688 and 9692, the cassette was inserted in the same orientation as the deleted gene(s) while in strain 0202 the cassette was oriented opposite to the deleted *ecfQ* gene (Figure 2). The deletion in strains 0202, 9688 and 9692 spans the genomic regions from position 1′134′763 to 1′135′446, 3′355′445 to 3′356′598 and 3′356′040 to 3′356′598, respectively.

Strain 15-02 (Δ[*ecfQ*, *ecfF*-*osrA*]) was constructed as follows: first, the kanamycin resistance cassette in strain 9688 was replaced by a spectinomycin/streptomycin resistance cassette (Ω) resulting in strain 9715. The cassette exchange was performed by conjugation into strain 9688 plasmid pRJ9715 whose insert corresponds to that of pRJ9688 with the kanamycin resistance cassette replaced by the Ω cassette inserted between the up- and downstream regions of the *ecfF*-*osrA* genes. Mutant strain 15-02 which is deleted for *ecfQ* and *ecfF*-*osrA* was obtained by using plasmid pRJ0202 to introduce the *ecfQ* deletion into strain 9715 via marker-exchange mutagenesis (Figure 2). The resulting deletions in strain 15-02 span the same genomic regions as in the individual mutants described above.

The Δblr3042 strain 0203 was constructed by a markerless in-frame deletion mutagenesis. This approach was chosen because tiling analysis of microarray data indicated that blr3042 is the promoter-proximal gene of a tricistronic operon consisting of blr3042, blr3043 and blr3044. Flanking regions of blr3042 were cloned into the suicide plasmid pK18*mobsacB* to yield plasmid pRJ0203. Plasmid pRJ0203 was transferred by conjugation from *E. coli* S17-1 to *B. japonicum* 110*spc*4. Kanamycin resistant exconjugants were selected and grown in the presence of 5% sucrose to force loss of the vector-encoded *sacB* gene. Resulting colonies were checked for kanamycin sensitivity, and the desired deletion was confirmed by PCR. In the resulting strain 0203 the genomic region from position 3′359′337 to 3′359′926 is deleted.

For complementation of strain 9692 (Δ*osrA*) with wild-type OsrA (resulting strain: 92-29) or mutant variants of OsrA (OsrA C129S: strain 92-36; OsrA C179S: strain 92-37; OsrA C129S: strain 92-38) respective plasmids were chromosomally integrated (see Table S5). Briefly, a 1′116-bp fragment containing the 3′ end of *ecfF* plus *osrA* (genome coordinates 3′355′542 to 3′356′646) was amplified by PCR using primer pairs listed in Table S6, cloned in the pGEM-T Easy vector, verified by sequencing and re-cloned into vector pSUP202pol4 to yield plasmid pRJ9729. To generate mutant versions of *osrA*, we used natural restriction sites within *osrA* (NcoI, AscI) and a PstI site at the 3′ end of *osrA*, which was incorporated via PCR. A 569-bp NcoI-PstI DNA fragment corresponding to the 3′ portion of *osrA* yet with *osrA* cysteine codons 129 and 179 mutated to TCC serine codons was synthesized (Eurofins MWG Operon, Ebersberg, Gemany). NcoI-PstI, AscI-PstI, or NcoI-AscI restriction fragments of the synthetic sequence were used to replace corresponding fragments in pRJ9729 resulting in plasmids pRJ9736 (OsrA C129S, C179S), pRJ9737 (OsrA C179S) and pRJ9738 (OsrA C129S).

Strain 92-30 served a control and contains vector pSUP202pol4 chromosomally inserted between *osrA* and bll3040. For its construction, a 451-bp fragment containing the 3′ end of bll3040 (genome coordinates 3′356′640 to 3′357′075) was PCR amplified using the primer pair listed in Table S6, cloned in the pGEM-T Easy vector, verified by sequencing and re-cloned in pSUP202pol4 resulting in plasmid pRJ9729. Plasmids pRJ9729, pRJ9736, pRJ9737, pRJ9738 and pRJ9730 were transformed into *E. coli* S17-1 and then mobilized by conjugation into *B. japonicum* strain 9692 as previously described [71] resulting in mutant strains 92-29, 92-36, 92-37, 92-38 and 92-30, respectively. The correct genomic structure (Figure 2) of the resulting strains was verified by PCR.

### DNA Work

Recombinant DNA work was performed according to standard protocols [72]. *B. japonicum* chromosomal DNA was isolated as described [71].

### Analyses of Stress Sensitivity

Zone inhibition assays were performed as described in [69]. The following compounds were tested at the indicated concentrations: H_2_O_2_ (10 mM, 100 mM, 1 M), diamide (10 mM, 100 mM, 1 M), FeSO_4_ (1 mM, 10 mM, 100 mM), *S*-nitroso-*N*-acetylpenicillamine (100 mM), S-nitrosoglutathione (100 mM), methylglyoxal (10 mM, 50 mM). Sensitivity to rose bengal was tested by spotting serial dilutions of bacteria from late exponential-phase cultures onto 1% PSY agar containing rose bengal (0.1 µM, 0.2 µM, 0.5 µM). Plates were illuminated with a tungsten light bulb (100 W, distance 95 cm, 2,000 lux) for 1 or 2 h and incubated in the dark four days at 30°C. Control plates were not exposed to light.

### Plant Growth Conditions and Inoculation

Soybean (*Glycine max* [L.] Merr. cv. Williams and cultivar „Green Butterbean“), mungbean (*Vigna radiata*) and cowpea (*Vigna unguiculata* [L.] Walp. cv. Red Caloona) seedlings were surface-sterilized as described [31], [71], [73], [74]. Determination of nitrogenase activity in bacteroids were performed as described previously [73].

### RNA Extraction and CDNA Synthesis

Harvest and storage of cells, RNA extraction and cDNA synthesis were done as previously described [68].

### Quantitative Real-time PCR

Expression of genes *ecfQ* and *ecfF* was analyzed by reverse transcription-based quantitative real-time PCR as previously described [41]. RNA was isolated from micro-oxically grown mid-log phase wild-type cells that were either untreated or exposed prior to harvest to one of the following treatments: 2 mM H_2_O_2_ for 10 min; 0.2 mM paraquat for 5 or 10 min; 0.5 µM rose bengal plus light exposure (20,000 lux) for 10 or 180 min; exposure to light for 60 min (control). Expression of the *ecfF* gene was analyzed in strains 92-29, 92-36, 92-37, 92-28 and 92-30 grown micro-oxically to mid-log phase, either untreated or exposed to 2 mM H_2_O_2_ for 10 min prior harvesting. cDNA (0.2 to 20 ng) in combination with 2.5 µM of primers pairs 1028-RT-F and 1028-RT-R or 3038-RT-F/3038-RT-R (Table S6) were used for monitoring expression of *ecfQ* and *ecfF*, respectively. The primary σ factor gene *sigA* was used as a reference for normalization (primers SigA-1155R and SigA-1069F; [41]). Data were evaluated by the method of Pfaffl [75].

### Primer Extension

The transcription start site of *ecfQ* and *ecfF* were determined as previously described [76], [77]. RNA was extracted from micro-oxically grown wild-type cells either non-stressed or treated with 2 mM H_2_O_2_ for 10 min. To determine transcription start site of *ecfQ* cDNAs were synthesized with primers pe-1028-1 or pe-1028-2 (Table S6). The same primers were used to obtain sequencing ladders from plasmid pRJ0211 (Table S5), containing the promoter and part of the *ecfQ* coding region. Likewise, primers pe-3038-1 and 3038-RT-R and plasmid pRJ9724 were used for determination of the transcription start site of *ecfF-osrA*.

### Microarrays

Global transcription levels were determined as described previously using a custom-designed Affymetrix chip [68], [69]. RNA template for cDNA synthesis was isolated from micro-oxically grown cells of the wild type and mutant strains 0202, 9688 and 9692, and also of H_2_O_2_-treated cells (2 mM, 10 min) for the wild type and two mutant strains (0202, 9688). For each strain and condition, a minimum of three biological replicates was prepared. RNA extraction, cDNA synthesis, fragmentation and labeling were done as described previously [68], [78]. GeneChip data analysis was performed using GeneSpring GX 7.3.1 software (Agilent). After filtering for probe sets which were called present or marginal in at least two out of three replicas, a statistical student *t*-test with a P*-*value threshold of 0.01 was applied. Genes were considered as differentially expressed if the fold-change value was <–3 or >+3 when comparing two strains or conditions. Data sets generated in this work are deposited in the GEO database under record number GSE39165.

### Bioinformatic Analyses

Phylogenetic analysis of *B. japonicum* ECF σ factors was conducted using MEGA version 4 [79]. For alignment of nucleotide and amino acid sequences, the T-COFFEE program was used (http://www.ebi.ac.uk/Tools/msa/tcoffee/ [80], [81]). Results were visualized with GeneDoc [82] and BioEdit [83]. Database searches for regulators that might bind to the up-stream region of *ecfQ* were done with the virtual footprint tool Prodoric (http://www.prodoric.de; [84]). Search for consensus motifs in EcfQ- and EcfF-target promoters was performed using the BioProspector suite (http://ai.stanford.edu/~xsliu/BioProspector/; [85]). DNA sequences corresponding to 200 bp located upstream of the promoter-proximal genes listed in Table 3 were used as input for the analysis. Parameters were set to search for a two-block motif with 5 nucleotides per block and a gap of 13 to 16 nucleotides between the blocks. Identified sequence motifs were aligned and visualized using the WebLogo tool (http://weblogo.berkeley.edu/; [86]). Genome-wide searches for putative EcfF target promoters focused on 200 bp regions upstream of genes or operons and were performed with the genome-scale DNA pattern search program from the RSAT collection of sequence analysis tools (http://rsat.ulb.ac.be/; [87]) Searches for amino acid sequence similarities were performed with BlastP (http://blast.ncbi.nlm.nih.gov/Blast.cgi?PAGE  =  Proteins). Topology prediction for OsrA was done with TOPCONS (http://topcons.cbr.su.se/; [88]). Protein localization prediction via a signal peptide search was performed using SignalP 4.0 (http://www.cbs.dtu.dk/services/SignalP/; [89]).

### Bacterial Two-hybrid System

For analysis of EcfF-OsrA interactions the BATCH system was used (Euromedex, Souffelweyersheim, France). Translational fusions of wild-type and mutant versions of OsrA to the C-terminal end of the T25 fragment of *Bordetella pertussis* adenylate cyclase (Cya) were generated by cloning of PCR-generated PstI-EcoRI fragments into vector pKT25 (resulting in plasmids pRJ9744, pRJ9752, pRJ9753, and pRJ9754; Table S5). Primers are listed in Table S6. For amplification of wild-type *osrA*, genomic DNA of *B. japonicum* 110*spc*4 was used while mutated *osrA* versions were amplified with plasmids pRJ9736, pRJ9737, or pRJ9738 as templates. In parallel, a translational fusion of EcfF to the C-terminal end of the Cya T18 fragment was generated. To do so, a PstI-XbaI fragment containing the wild-type *ecfF* gene was amplified (primer pair listed in Table S6, genomic *B. japonicum* DNA as template) and cloned into vector pUT18C yielding plasmid pRJ9746. All constructed plasmids were verified by sequencing. To study interaction of EcfF with different versions of OsrA, *E. coli* strain BTH101 was co-transformed with pRJ9746 and one of the plasmids expressing a T25-OsrA fusion. For β-galactosidase activity assays, co-transformed clones were inoculated into 6 ml LB medium containing appropriate antibiotics and 0.5 mM IPTG (isopropyl β-D-1-thiogalactopyranonoside). Cultures were grown for 18 h at 30°C, and aliquot(s) from 50 µl to 200 µl were used to determine β-galactosidase activity as described elsewhere [43].

## Supporting Information

Figure S1 **Alignment of OsrA-homologs.** Numbers on the right of each line refer to the position of the last amino acid within the corresponding protein sequence. Shaded in black, dark grey, and light grey are nucleotides which are identical in all, 80%, and 60% of the sequences, respectively. Arrowheads indicate conserved cysteines. GI numbers of the proteins are as follows: *Bradyrhizobium japonicum* USDA 110 (*BJ*) OsrA - 81738347 and TmrS (Blr4929) - 81736761, *Mesorhizobium loti* MAFF303099 (*ML*) 81779508, *Agrobacterium tumefaciens* str. C58 (*AT*) 15889542, *Rhizobium etli* CFN 42 (*RE*) 123508957, *Sinorhizobium meliloti* 1021 (*SM*) 81813033, *Burkholderia pseudomallei* (*BP*) 81379776, *Pseudomonas putida* KT2440 (*PP*) 81442010, *Dechloromonas aromatica* RCB (*DA*) Daro_1521–71907153 and Daro_2589–71908203.
(TIF)

Figure S2 **Venn diagram of H_2_O_2_-responsive genes in the** ***B. japonicum*** **wild-type strain and the regulons of ECF σ factors EcfQ and EcfF.** Hydrogen peroxide-responsive genes were identified by transcriptome analyses of untreated wild-type cells with cells exposed to 2 mM H_2_O_2_ for 10 min. Similarly, regulons of EcfQ and EcfF were determined by comparing the transcriptome of Δ*ecfQ* and Δ(*ecfF*-*osrA*) mutant strains, respectively, both treated with 2 mM H_2_O_2_ for 10 min, with identically stressed wild-type cells. All strains we grown micro-oxically. Size and overlap of the regulons are drawn to scale with numbers of differentially expressed genes (3-fold change cut-off) indicated in the respective segments. Total number and numbers of down- (↓) and up-regulated genes (↑) are shown next to individual regulons.
(TIF)

Table S1 **List of 225** ***B. japonicum*** **genes which are differentially expressed after treatment with 2 mM H_2_O_2_ for 10 min in wild-type cells grown micro-oxically in PSY medium as compared to untreated wild-type cells.**
(DOCX)

Table S2 **List of** ***B. japonicum*** **genes differentially expressed in the Δ** ***ecfQ*** **strain 0202 compared to the wild type.** Cells were grown micro-oxically and harvested after no further treatment (**A**) or after exposure to 2 mM H_2_O_2_ for 10 min (**B**).
(DOCX)

Table S3 **List of** ***B. japonicum*** **genes differentially expressed in the Δ(** ***ecfF*** **-** ***osrA*** **) strain 9688 compared to the wild type.** Cells were grown micro-oxically and harvested after no further treatment (**A**) or after exposure to 2 mM H_2_O_2_ for 10 min (**B**).
(DOCX)

Table S4 **List of** ***B. japonicum*** **genes differentially expressed in micro-oxically grown cells of the Δ** ***osrA*** **mutant strain 9692 compared to the wild type.**
(DOCX)

Table S5 **Bacterial strains and plasmids used in this work.**
(DOCX)

Table S6 **Primers used in this study.**
(DOCX)
