# Supplementary figures and images for: Reactive Oxygen Species-Inducible ECF σ Factors of Bradyrhizobium japonicum
Source: PLoS One. 2012 Aug 16;7(8):e43421. doi: 10.1371/journal.pone.0043421 (PMC3420878; doi:10.1371/journal.pone.0043421)

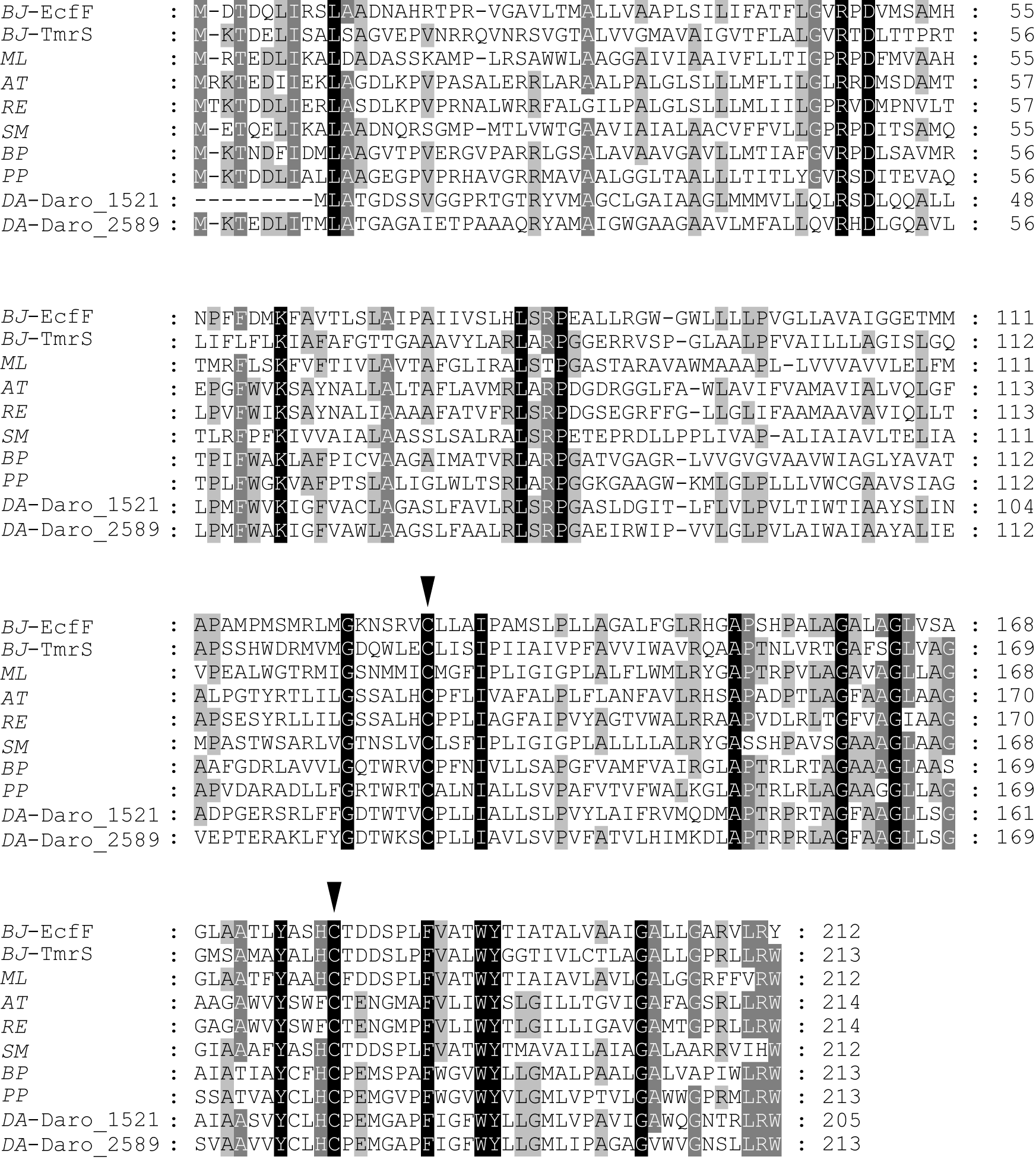

Supplement: Figure S1 — Alignment of OsrA-homologs. Numbers on the right of each line refer to the position of the last amino acid within the corresponding protein sequence. Shaded in black, dark grey, and light grey are nucleotides which are identical in all, 80%, and 60% of the sequences, respectively. Arrowheads indicate conserved cysteines. GI numbers of the proteins are as follows: Bradyrhizobium japonicum USDA 110 (BJ) OsrA - 81738347 and TmrS (Blr4929) - 81736761, Mesorhizobium loti MAFF303099 (ML) 81779508, Agrobacterium tumefaciens str. C58 (AT) 15889542, Rhizobium etli CFN 42 (RE) 123508957, Sinorhizobium meliloti 1021 (SM) 81813033, Burkholderia pseudomallei (BP) 81379776, Pseudomonas putida KT2440 (PP) 81442010, Dechloromonas aromatica RCB (DA) Daro_1521–71907153 and Daro_2589–71908203. (TIF) [file pone.0043421.s001.tif]

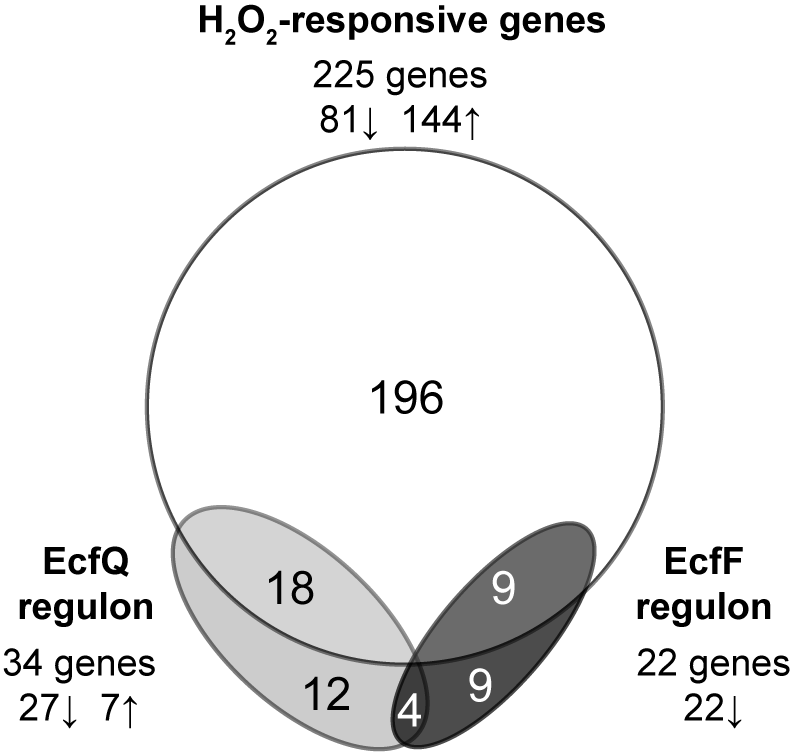

Supplement: Figure S2 — Venn diagram of H2O2-responsive genes in the B. japonicum wild-type strain and the regulons of ECF σ factors EcfQ and EcfF. Hydrogen peroxide-responsive genes were identified by transcriptome analyses of untreated wild-type cells with cells exposed to 2 mM H2O2 for 10 min. Similarly, regulons of EcfQ and EcfF were determined by comparing the transcriptome of ΔecfQ and Δ(ecfF-osrA) mutant strains, respectively, both treated with 2 mM H2O2 for 10 min, with identically stressed wild-type cells. All strains we grown micro-oxically. Size and overlap of the regulons are drawn to scale with numbers of differentially expressed genes (3-fold change cut-off) indicated in the respective segments. Total number and numbers of down- (↓) and up-regulated genes (↑) are shown next to individual regulons. (TIF) [file pone.0043421.s002.tif]
