# Supplementary material for: Reactive Oxygen Species-Inducible ECF σ Factors of Bradyrhizobium japonicum
Source: PLoS One. 2012 Aug 16;7(8):e43421. doi: 10.1371/journal.pone.0043421 (PMC3420878; doi:10.1371/journal.pone.0043421)
Supplement: Table S1 — List of 225 B. japonicum genes which are differentially expressed after treatment with 2 mM H2O2 for 10 min in wild-type cells grown micro-oxically in PSY medium as compared to untreated wild-type cells. (DOCX) [file pone.0043421.s003.docx]

**Table S1.** List of 225 *B. japonicum* genes which are differentially expressed after treatment with 2 mM H_2_O_2_ for 10 min in wild-type cells grown micro-oxically in PSY medium as compared to untreated wild-type cells^a^.

| **Gene no.^b^** | **Fold change** | **Known or predicted function of gene product^c^** |
| --- | --- | --- |
| **Transcriptional regulators** | | |
| bll1028 | 34.8 | σ factor EcfQ |
| blr3420 | 30.9 | transcriptional regulatory protein PadR-like family |
| blr2577 | 20 | transcriptional regulatory protein TetR family |
| bll2775 | 19.6 | transcriptional regulatory protein LysR family |
| bll4010 | 15.9 | transcriptional regulatory protein PadR-like family |
| blr3038 | 14.4 | σ factor EcfF |
| blr0736 | 14.4 | transcriptional regulatory protein MarR family |
| bll2319 | 13.8 | transcriptional regulatory protein GntR family |
| blr4013 | 10.6 | transcriptional regulatory protein MarR family |
| blr3963 | 10.3 | transcriptional regulatory protein LysR family |
| blr3814 | 8.1 | transcriptional regulatory protein Crp family |
| bll1150 | 6.3 | transcriptional regulatory protein LysR family |
| blr5345 | 6.1 | transcriptional regulatory protein ArsR family |
| bll2604 | 5.2 | transcriptional regulatory protein MarR family |
| bll5689 | 5.2 | transcriptional regulatory protein TetR family |
| blr5122 | 4.7 | transcriptional regulatory protein TetR family |
| blr4826 | 4.4 | LexA repressor |
| blr0347 | 4.2 | transcriptional regulatory protein MarR family |
| blr6277 | 4.1 | transcriptional regulatory protein GntR family |
| blr8125 | 3.4 | transcriptional regulatory protein AsnC family |
| blr3952 | 3.3 | transcriptional regulatory protein TetR family |
| bll3916 | 3 | transcriptional regulatory protein LacI family |
| blr1883 | -3 | RNA polymerase sigma-54 subunit |
| blr1880 | -3.1 | transcriptional regulatory protein LuxR family |
| blr7666 | -3.2 | transcriptional regulatory protein AraC family |
| bll4976 | -3.4 | transcriptional regulatory protein MarR family |
| bll5886 | -3.4 | two-component hybrid sensor and regulator |
| bll2094 | -3.9 | transcriptional regulatory protein GntR family |
| blr1216 | -7.2 | transcriptional regulatory protein Ferric uptake regulator family |
| **Other categories** | | |
| bll3418 | 205 | putative epoxide hydrolase 1 (EC 3.3.2.3) |
| bll4012 | 53.8 | organic hydroperoxide resistance protein |
| bll0735 | 43.6 | organic hydroperoxide resistance protein |
| blr6636 | 30.3 | ATP synthase subunit |
| blr2776 | 29.4 | putative patatin-like phospholipase |
| bll7849 | 16.4 | putative glutathione S-transferase |
| bll7983 | 15.7 | glutathione transferase |
| bll3948 | 13.7 | monocarboxylate MFS permease |
| blr2578 | 9.6 | MFS permease |
| blr0778 | 9.6 | catalase |
| bll4280 | 9.1 | probable ThiJ-PfpI family protein |
| blr7043 | 8.5 | peptide methionine sulfoxide reductase |
| bll1027 | 7.8 | putative cytochrome *c* biogenesis protein |
| bll5865 | 7.6 | putative multidrug resistance protein |
| bll5855^d^ | 7.5 | peptide methionine sulfoxide reductase |
| bll7034 | 6.7 | MDO-like protein |
| bll5121 | 5.7 | major facilitator superfamily transporter |
| bll0339 | 5.5 | 4-hydroxyphenylpyruvate dioxygenase (EC 1.13.11.27) |
| bll7217 | 5.4 | probable site-specific integrase-recombinase |
| *bll3903* | 5 | putative multidrug resistance protein |
| *bll3902* | 3.6 | AcrB-AcrD-AcrF family protein |
| blr2826 | 5 | dihydroxy-acid dehydratase |
| blr5698 | 4.4 | similar to protein-export membrane protein SecD |
| blr7431 | 4.2 | excinuclease ABC subunit B |
| *blr2489* | 3.9 | anthranilate synthase component I and II |
| *blr2490* | 3.3 | adenine phosphoribosyltransferase |
| bll0346 | 3.8 | putative oxidoreductase |
| blr3130 | 3.8 | serine protease DO-like precursor |
| blr7466 | 3.8 | ribonuclease |
| bll7427 | 3.7 | probable ligninase |
| bll5755 | 3.5 | RecA protein |
| bll4731 | 3.4 | probable threonine dehydratase (EC 4.2.1.16) |
| blr8158 | 3.3 | murein endopeptidase |
| blr6637 | 3.3 | putative cytochrome *c* |
| bll0414 | 3.2 | 3-isopropylmalate dehydrogenase |
| blr1535 | 3.2 | probable holliday junction nuclease |
| bll1418 | 3.1 | methionine synthase |
| blr2322 | 3.1 | citrate utilization protein B |
| blr2696 | 3 | cytochrome *c* peroxidase |
| bll7906 | 3 | putative ferredoxin |
| blr1727 | -3 | HupH protein homolog |
| blr5827 | -3.1 | flagellar basal-body rod protein |
| blr1964 | -3.1 | putative sugar hydrolase |
| blr1756 | -3.3 | nitrogenase metalloclusters biosynthesis protein |
| blr4211 | -3.3 | putative cell division inhibitor protein |
| bsl5256 | -3.3 | probable polar flagellar motor switch protein |
| bsl5811 | -3.3 | flagellar biosynthetic protein |
| bll0718 | -3.3 | putative transporter |
| blr4932 | -3.3 | putative cation efflux system protein |
| blr1755 | -3.4 | *R. etli iscN* homolog |
| blr7922 | -3.5 | ABC transporter substrate-binding protein |
| blr1759 | -3.6 | FeMo cofactor biosynthesis protein |
| blr7759 | -3.6 | ornithine decarboxylase |
| blr2143 | -3.6 | similar to cytochrome P450-family protein |
| blr6579 | -3.6 | ABC transporter ATP-binding protein |
| blr0162 | -3.6 | 50S ribosomal protein L28 |
| bll1906 | -3.8 | N-acetyltransferase NrgA homolog |
| bll6680 | -3.9 | bacterioferritin |
| bll6950 | -4 | putative pyrophosphorylase (EC 2.4.2.-) |
| blr2036 | -4 | oxidoreductase |
| bll2388 | -4 | cytochrome *c*_2_ |
| blr1719 | -4.2 | molybdenum transport system permease protein |
| bll2007 | -4.9 | coproporphyrinogen III dehydrogenase |
| blr2131 | -5 | probable oxygenase |
| blr2106 | -5.8 | L-ectoine synthase |
| *bll5814* | -6.4 | probable flagellar basal-body rod protein |
| *bll5813* | -7.1 | flagellar basal-body rod protein |
| *bll5812* | -3.7 | flagellar hook-basal body complex protein |
| **Hypothetical proteins and proteins of unknown function** | | |
| bll3417 | 193.6 | hypothetical protein |
| *bll7429* | 131.5 | unknown protein |
| *bsl7428* | 173.2 | hypothetical protein |
| bll2772 | 129.8 | unknown protein |
| bll3419 | 98.3 | hypothetical protein |
| bll3416 | 74.7 | unknown protein |
| bll4011 | 71.1 | hypothetical protein |
| bll5457 | 49.3 | hypothetical protein |
| blr7542 | 47.2 | unknown protein |
| bsr4694 | 38.4 | unknown protein |
| bll2771 | 36.4 | hypothetical protein |
| blr0349 | 20.5 | unknown protein |
| blr4067 | 19.7 | hypothetical protein |
| blr2773 | 19.5 | unknown protein |
| blr7741 | 19.3 | hypothetical protein |
| bll1068 | 19.1 | hypothetical protein |
| bsl0348 | 16.4 | unknown protein |
| *bll5344* | 16.4 | hypothetical protein |
| *bll5343* | 5.2 | hypothetical protein |
| blr2774 | 15.8 | hypothetical protein |
| bsl5107 | 15.7 | unknown protein |
| *bll0506* | 14 | hypothetical protein |
| *bll0505* | 15.5 | hypothetical protein |
| blr2500 | 13.6 | hypothetical protein |
| blr7943 | 13.5 | hypothetical protein |
| bsl7850 | 12.2 | unknown protein |
| blr1151 | 11.5 | hypothetical protein |
| bll1026 | 11.4 | hypothetical protein |
| *blr2320* | 11.2 | hypothetical protein |
| *blr2321* | 6.6 | hypothetical protein |
| bll3504 | 11.2 | unknown protein |
| bll2645 | 11.1 | hypothetical protein |
| bsr4431 | 10.7 | hypothetical protein |
| bll0176 | 10.5 | unknown protein |
| bll4461 | 10.3 | unknown protein |
| bsl5486 | 8.8 | unknown protein |
| blr3039 | 8.1 | anti-σ factor OsrA |
| blr3596 | 8 | hypothetical protein |
| blr7338 | 7.7 | hypothetical protein |
| blr4673 | 7.7 | hypothetical protein |
| bll2701 | 7.1 | unknown protein |
| blr2519 | 7 | hypothetical protein |
| bll2595 | 6 | unknown protein |
| bsr2594 | 5.6 | unknown protein |
| bsl4436 | 5.4 | unknown protein |
| bsl2593 | 5.4 | hypothetical protein |
| blr0321 | 5.4 | unknown protein |
| blr0485 | 5.4 | hypothetical protein |
| blr4468 | 5.3 | unknown protein |
| bll5259 | 5.3 | hypothetical protein |
| bsl3813 | 5.2 | unknown protein |
| bsl4436 | 4.9 | unknown protein |
| bll1305 | 4.7 | unknown protein |
| bll0734 | 4.7 | hypothetical protein |
| bll6527 | 4.7 | hypothetical protein |
| bll0661 | 4.4 | hypothetical protein |
| bll5329 | 4.4 | hypothetical protein |
| blr2827 | 4 | hypothetical protein |
| bsr7111 | 4 | unknown protein |
| blr8110 | 3.9 | hypothetical protein |
| bll2845 | 3.9 | unknown protein |
| blr0354 | 3.8 | hypothetical protein |
| bll4712 | 3.8 | unknown protein |
| blr1018 | 3.7 | hypothetical protein |
| bll7128 | 3.6 | unknown protein |
| bsl3012 | 3.4 | hypothetical protein |
| bll3594 | 3.4 | hypothetical protein |
| blr0248 | 3.4 | unknown protein |
| bsl4593 | 3.3 | unknown protein |
| bll3089 | 3.3 | unknown protein |
| bll0555 | 3.2 | hypothetical protein |
| blr4680 | 3.2 | hypothetical protein |
| bsl6617 | 3.2 | unknown protein |
| bsr7045 | 3.2 | hypothetical protein |
| bll0839 | 3.1 | hypothetical protein |
| blr4562 | 3.1 | unknown protein |
| blr7005 | 3.1 | hypothetical protein |
| blr2777 | 3 | hypothetical protein |
| blr5947 | 3 | unknown protein |
| bsl2206 | 3 | hypothetical protein |
| bsl4014 | 3 | unknown protein |
| bll7907 | 3 | hypothetical protein |
| bsl1870 | -3 | unknown protein |
| bll5843 | -3 | hypothetical protein |
| blr2668 | -3 | hypothetical protein |
| bll7538 | -3.1 | hypothetical protein |
| bsr1758 | -3.1 | unknown protein |
| blr7050 | -3.1 | unknown protein |
| bsr7087 | -3.1 | unknown protein |
| blr7502 | -3.1 | unknown protein |
| bsr3073 | -3.2 | hypothetical protein |
| blr5768 | -3.2 | unknown protein |
| bsl2070 | -3.2 | hypothetical protein |
| bll7394 | -3.2 | hypothetical protein |
| bll5520 | -3.2 | hypothetical protein |
| blr1867 | -3.3 | hypothetical protein |
| bll0737 | -3.4 | hypothetical protein |
| bll5679 | -3.4 | hypothetical protein |
| bsl4522 | -3.4 | unknown protein |
| blr2044 | -3.4 | unknown protein |
| blr4624 | -3.4 | hypothetical protein |
| bll1980 | -3.4 | hypothetical protein |
| blr1879 | -3.5 | hypothetical protein |
| bll7386 | -3.5 | unknown protein |
| blr1433 | -3.7 | hypothetical protein |
| blr2975 | -3.8 | hypothetical protein |
| blr1992 | -3.8 | unknown protein |
| bll6468 | -3.9 | hypothetical protein |
| blr4988 | -4 | unknown protein |
| *blr1850* | -4.2 | unknown protein |
| *blr1851* | -3.3 | unknown protein |
| blr1954 | -4.4 | unknown protein |
| bll6909 | -4.5 | hypothetical protein |
| bll6577 | -4.7 | hypothetical protein |
| bll7405 | -4.8 | hypothetical protein |
| blr4174 | -5.2 | hypothetical protein |
| bsr4175 | -5.2 | hypothetical protein |
| bsl2574 | -5.7 | unknown protein |
| bll1767 | -5.7 | hypothetical protein |
| blr1130 | -5.8 | hypothetical protein |
| blr1726 | -5.9 | unknown protein |
| *bsr2010* | -6.1 | unknown protein |
| *blr2011* | -5.2 | unknown protein |
| bsr1907 | -6.1 | unknown protein |
| bll1981 | -6.2 | hypothetical protein |
| bll2085 | -6.5 | hypothetical protein |
| blr8234 | -6.5 | unknown protein |
| bll3193 | -8.7 | unknown protein |

^a^ Differentially expressed genes were selected based on a 3-fold change cut-off.

^b^ Nomenclature according to Kaneko et al., 2002. Numbers of genes organized in putative operons are indicated in italics with co-transcribed promoter-distal genes indented to the right.

^c^ Gene description according to Kaneko et al., 2002 with modifications.

^d^ bll5855 is annotated by Kaneko et al., 2002 as a hypothetical protein. BLAST analysis indicated that it codes for a conserved domain (MsrB) present in peptide methionine sulfoxide reductases.

Kaneko T, Nakamura Y, Sato S, Minamisawa K, Uchiumi T, et al. (2002) Complete genomic sequence of nitrogen-fixing symbiotic bacterium *Bradyrhizobium japonicum* USDA110. DNA Res 9: 189-197.
