# Supplementary material for: Reactive Oxygen Species-Inducible ECF σ Factors of Bradyrhizobium japonicum
Source: PLoS One. 2012 Aug 16;7(8):e43421. doi: 10.1371/journal.pone.0043421 (PMC3420878; doi:10.1371/journal.pone.0043421)
Supplement: Table S2 — List of B. japonicum genes differentially expressed in the Δ ecfQ strain 0202 compared to the wild type. Cells were grown micro-oxically and harvested after no further treatment (A) or after exposure to 2 mM H2O2 for 10 min (B). (DOCX) [file pone.0043421.s004.docx]

**Table S2.** List of *B. japonicum* genes differentially expressed in the Δ*ecfQ* strain compared to the wild type. Cells were grown micro-oxically and harvested after no further treatment (**A**) or after exposure to 2 mM H_2_O_2_ for 10 min (**B**).^a^

**A**

| **Gene no.^b^** | **Fold change** | **Known or predicted gene product^c^** |
| --- | --- | --- |
| blr1214 | 8.1 | putative lipoprotein |
| bll6888 | 7.9 | putative porin |
| bll3735 | 3.8 | putative outer-membrane immunogenic protein precursor |
| blr2455 | 3.1 | isocitrate lyase (EC 4.1.3.1) |
| bsl1637 | -3.2 | unknown protein |
| bll1858 | -3.3 | hypothetical protein |
| bll2004 | -3.3 | unknown protein |
| blr8234 | -3.4 | unknown protein |
| bll1028 | -24.6 | σ factor EcfQ |

**B**

| **Gene no.^b^** | **Fold change** | **Known or predicted gene product^c^** |
| --- | --- | --- |
| blr1215 | 4.6 | hypothetical protein |
| bll0342 | 4.4 | fumarylacetoacetase (EC 3.7.1.2) |
| bsr6573 | 4.0 | unknown protein |
| blr5231 | 3.8 | sigma32-like factor |
| bll6888 | 3.7 | putative porin |
| blr6572 | 3.3 | unknown protein |
| bll0343 | 3.2 | homogentisate 1,2-dioxygenase |
| bll0333 | -3.0 | probable alcohol dehydrogenase precursor |
| blr0337 | -3.0 | putative carbon monoxide dehydrogenase medium chain (EC 1.2.99.2) |
| trnN-GUU-1 | -3.1 | tRNA-Asn(GGT) |
| blr4468 | -3.2 | unknown protein |
| blr2519 | -3.3 | hypothetical protein |
| bll2494 | -3.3 | hypothetical protein |
| bll4712 | -3.4 | unknown protein |
| bll2845 | -3.4 | unknown protein |
| bll0331 | -3.5 | two-component response regulator |
| blr0321 | -3.8 | unknown protein |
| bll4173 | -3.8 | unknown protein |
| blr3534 | -3.8 | putative carbon monoxide dehydrogenase medium chain (EC 1.2.99.2) |
| bll0332 | -4.4 | unknown protein |
| blr0354 | -4.5 | hypothetical protein |
| blr5698 | -4.7 | similar to protein-export membrane protein SecD |
| blr2520 | -4.8 | hypothetical protein |
| bll3594 | -5.1 | hypothetical protein |
| bll1305 | -5.2 | unknown protein |
| blr7338 | -5.3 | hypothetical protein |
| bsl5107 | -9.3 | unknown protein |
| blr3596 | -9.5 | hypothetical protein |
| bll0176 | -9.9 | unknown protein |
| blr0349 | -12.2 | unknown protein |
| bsl0348 | -12.7 | unknown protein |
| bll2645 | -13.5 | hypothetical protein |
| blr7943 | -38.8 | hypothetical protein |
| bll1028 | -203.3 | σ factor EcfQ |

^a^ Differentially expressed genes were selected based on a 3-fold change cut-off.

^b^ Nomenclature according to Kaneko et al., 2002.

^c^ Gene description according to Kaneko et al., 2002.

Kaneko T, Nakamura Y, Sato S, Minamisawa K, Uchiumi T, et al. (2002) Complete genomic sequence of nitrogen-fixing symbiotic bacterium *Bradyrhizobium japonicum* USDA110. DNA Res 9: 189-197.
