# Supplementary material for: Reactive Oxygen Species-Inducible ECF σ Factors of Bradyrhizobium japonicum
Source: PLoS One. 2012 Aug 16;7(8):e43421. doi: 10.1371/journal.pone.0043421 (PMC3420878; doi:10.1371/journal.pone.0043421)
Supplement: Table S3 — List of B. japonicum genes differentially expressed in the Δ( ecfF - osrA ) strain 9688 compared to the wild type. Cells were grown micro-oxically and harvested after no further treatment (A) or after exposure to 2 mM H2O2 for 10 min (B). (DOCX) [file pone.0043421.s005.docx]

**Table S3.** List of *B. japonicum* genes differentially expressed in the Δ*ecfF*-*osrA* strain compared to the wild type. Cells were grown micro-oxically and harvested after no further treatment (**A**) or after exposure to 2 mM H_2_O_2_ for 10 min (**B**).^a^

**A**

| **Gene no.^b^** | **Fold change** | **Known or predicted gene product^c^** |
| --- | --- | --- |
| blr0149 | 3.3 | cytochrome *o* ubiquinol oxidase subunit II |
| bll6876 | 3.1 | flagellar basal-body rod protein |
| bll0148 | 3.1 | MFS permease |
| *blr3038* | -7.7 | σ factor EcfF |
| *blr3039* | -64.9 | anti-σ factor OsrA |

**B**

| **Gene no.^b^** | **Fold change** | **Known or predicted gene product^c^** |
| --- | --- | --- |
| bll2542 | -3.0 | quinolinate synthetase A |
| bll5259 | -3.1 | hypothetical protein |
| *blr7489* | -3.1 | lactoylglutathione lyase |
| *blr7490* | -3.2 | hypothetical protein |
| *blr7491* | -3.6 | putative 2-keto-gluconate dehydrogenase |
| blr0335 | -3.2 | putative carbon monoxide dehydrogenase small chain |
| blr2489 | -3.3 | anthranilate synthase component I and II |
| bll6527 | -4.7 | hypothetical protein |
| blr0336 | -4.7 | carbon monoxide dehydrogenase large chain |
| bll4784 | -4.8 | aldehyde dehydrogenase |
| blr0337 | -5.0 | putative carbon monoxide dehydrogenase medium chain (EC 1.2.99.2) |
| *bll0333* | -5.0 | probable alcohol dehydrogenase precursor |
| *bll0332* | -9.3 | unknown protein |
| bll0331 | -5.5 | two-component response regulator |
| bll5855^d^ | -6.2 | peptide methionine sulfoxide reductase |
| blr7043 | -7.0 | peptide methionine sulfoxide reductase |
| *bll1027* | -15.4 | putative cytochrome *c* biogenesis protein |
| *bll1026* | -20.1 | hypothetical protein |
| bsr4431 | -15.9 | hypothetical protein |
| blr7741 | -28.3 | hypothetical protein |
| *blr3038* | -60.6 | σ factor EcfF |
| *blr3039* | -210.1 | anti-σ factor OsrA |

^a^ Differentially expressed genes were selected based on a 3-fold change cut-off.

^b^ Nomenclature according to Kaneko et al., 2002. Numbers of genes organized in putative operons are indicated in italics with co-transcribed promoter-distal genes indented to the right.

^c^ Gene description according to Kaneko et al., 2002 with modifications.

^d^ bll5855 is annotated by Kaneko et al., 2002 as a hypothetical protein. BLAST analysis indicated that it codes for a conserved domain (MsrB) present in peptide methionine sulfoxide reductases.

Kaneko T, Nakamura Y, Sato S, Minamisawa K, Uchiumi T, et al. (2002) Complete genomic sequence of nitrogen-fixing symbiotic bacterium *Bradyrhizobium japonicum* USDA110. DNA Res 9: 189-197.
