# Supplementary material for: Reactive Oxygen Species-Inducible ECF σ Factors of Bradyrhizobium japonicum
Source: PLoS One. 2012 Aug 16;7(8):e43421. doi: 10.1371/journal.pone.0043421 (PMC3420878; doi:10.1371/journal.pone.0043421)
Supplement: Table S4 — List of B. japonicum genes differentially expressed in micro-oxically grown cells of the Δ osrA mutant strain 9692 compared to the wild type. (DOCX) [file pone.0043421.s006.docx]

**Table S4.** List of *B. japonicum* genes differentially expressed in micro-oxically grown cells of the Δ*osrA* mutant strain compared to the wild type.^a^

| **Gene no.^b^** | **Fold change** | **Known or predicted gene product^c^** |
| --- | --- | --- |
| *bll1027* | 89.5 | putative cytochrome *c* biogenesis protein |
| *bll1026* | 92.4 | hypothetical protein |
| bll6527 | 78.3 | hypothetical protein |
| blr7741 | 54.2 | hypothetical protein |
| bll5855^d^ | 20.6 | peptide methionine sulfoxide reductase |
| bsr4431 | 20.0 | hypothetical protein |
| *blr3038* | 18.8 | σ factor EcfF |
| *blr3039* | -19.9 | anti-σ factor OsrA |
| *bll0506* | 16.6 | hypothetical protein |
| *bll0505* | 26.6 | hypothetical protein |
| *blr7043* | 13.5 | peptide methionine sulfoxide reductase |
| *blr7044* | 5.4 | peptide methionine sulfoxide reductase |
| blr2776 | 13.1 | putative patatin-like phospholipase |
| bll1025 | 10.9 | unknown protein |
| blr7434 | 5.8 | hypothetical protein |
| bll0507 | 5.6 | hypothetical protein |
| blr1349 | 4.9 | hypothetical protein |
| bsl4407 | 4.7 | unknown protein |
| blr7936 | 4.6 | hypothetical protein |
| blr0834 | 4.2 | peptide methionine sulfoxide reductase |
| bsr7045 | 4.2 | hypothetical protein |
| *bll6529* | 4.1 | unknown protein |
| *bsl6528* | 3.0 | hypothetical protein |
| blr6167 | 4.0 | unknown protein |
| blr0305 | 3.9 | unknown protein |
| blr4928 | 3.8 | ECF σ factor |
| bll2508 | 3.8 | hypothetical glutathione S-transferase like protein |
| bll3384 | 3.8 | ABC transporter ATP-binding protein |
| bll7811 | 3.8 | hypothetical protein |
| bll1466 | 3.4 | unknown protein |
| blr1469 | 3.3 | hypothetical protein |
| blr3677 | 3.3 | putative monooxygenase component |
| blr3678 | 3.2 | putative oxidoreductase |
| bsl1473 | 3.2 | hypothetical protein |
| bsr5508 | 3.1 | peptide methionine sulfoxide reductase |
| blr3680 | 3.1 | hypothetical protein |
| bll0304 | 3.1 | two-component response regulator |
| bll6454 | -3.0 | ABC transporter permease protein |
| blr4463 | -3.3 | probable ABC transporter substrate-binding protein |

^a^ Differentially expressed genes were selected based on a 3-fold change cut-off.

^b^ Nomenclature according to Kaneko et al., 2002. Numbers of genes organized in putative operons are indicated in italics with co-transcribed promoter-distal genes indented to the right.

^c^ Gene description according to Kaneko et al., 2002 with modifications.

^d^ bll5855 is annotated by Kaneko et al., 2002 as a hypothetical protein. BLAST analysis indicated that it codes for a conserved domain (MsrB) present in peptide methionine sulfoxide reductases.

Kaneko T, Nakamura Y, Sato S, Minamisawa K, Uchiumi T, et al. (2002) Complete genomic sequence of nitrogen-fixing symbiotic bacterium *Bradyrhizobium japonicum* USDA110. DNA Res 9: 189-197.
