# Supplementary material for: Reactive Oxygen Species-Inducible ECF σ Factors of Bradyrhizobium japonicum
Source: PLoS One. 2012 Aug 16;7(8):e43421. doi: 10.1371/journal.pone.0043421 (PMC3420878; doi:10.1371/journal.pone.0043421)
Supplement: Table S5 — Bacterial strains and plasmids used in this work. (DOCX) [file pone.0043421.s007.docx]

**Table S5.** Bacterial strains and plasmids used in this work.

| **Strain or plasmid** | **Relevant genotype or phenotype** | **Source / Reference** |
| --- | --- | --- |
| ***E. coli* strains** | | |
| DH5α | *supE44* Δ*lacU169* (φ80 *lacZ*ΔM15) *hsdR17 recA1*  *gyrA96 thi-1 relA2* | BRL, Gaithersburg, USA |
| S17-1 | Sm^r^ Sp^r^ *hsdR* (RP4-2 *kan*::Tn7 *tet*::Mu; integrated into the chromosome) | Simon et al., 1983 |
| BTH101 | F^-^ *cya-99 araD139 galE15 galK16 rpsL1 (Str^r^) hsdR2 mcrA1 mcrB1* | Euromedex, Souffelweyersheim, France |
| ***B. japonicum* strains** | | |
| 110*spc*4 | Sp^r^ wild type | Regensburger and Hennecke 1983 |
| 0202 | Sp^r^ Km^r^ Δ*ecfQ*::*aphII* (opposite orientation) | This work |
| 0203 | Sp^r^ Δblr3042 | This work |
| 9688 | Sp^r^ Km^r^ Δ(*ecfF-osrA*)::*aphII* (same orientation) | This work |
| 9715 | Sp^r^ Str^r^ Δ(*ecfF-osrA*)::Ω (same orientation) | This work |
| 9692 | Sp^r^ Km^r^ Δ*osrA*::*aphII* (same orientation) | This work |
| 15-02 | Sp^r^ Str^r^ Km^r^ Δ(*ecfF-osrA*)::Ω (same orientation), Δ*ecfQ*::*aphII* (opposite orientation) | This work |
| 92-29 | Sp^r^ Km^r^ Tet^r^ wild-type *osrA* chromosomally integrated into 9692 | This work |
| 92-30 | Sp^r^ Km^r^ Tet^r^ pSUP202pol4 chromosomally integrated into 9692 | This work |
| 92-36 | Sp^r^ Km^r^ Tet^r^ *osrA* coding for OsrA C129S+C179S chromosomally integrated into strain 9692 | This work |
| 92-37 | Sp^r^ Km^r^ Tet^r^ *osrA* coding for OsrA C179S chromosomally integrated into strain 9692 | This work |
| 92-38 | Sp^r^ Km^r^ Tet^r^ *osrA* coding for OsrA C129S chromosomally integrated into strain 9692 | This work |
| **Plasmids** | | |
| pGEM-T Easy | Ap^r^ cloning vector | Promega, Madison, WI, USA |
| pBluescript SK(+) | Ap^r^ cloning vector | Stratagene, La Jolla, CA, USA |
| pBSL86 | Ap^r^ Km^r^ | Alexeyev et al., 1995 |
| pBSL15Ω | Ap^r^ Sp^r^ Str^r^ | Lindemann et. al., 2010 |
| pSUP202pol4 | Tc^r^ (pSUP202) part of the polylinker from pBluescript II KS(+) between EcoRI and PstI | Fischer et al., 1993 |
| pK18*mobsacB* | Km^r^ mobilizable pUC18 derivative, *mob*, *sacB* | Schäfer et. al, 1994 |
| pKT25 | Km^r^ expression vector, used to create translational fusion of the T25 fragment (the first 224 amino acids of CyaA) at the N-terminus of a protein | Euromedex, Souffelweyersheim, France |
| pUT18C | Ap^r^ expression vector, used to create translational fusion of the T18 fragment (amino acids 225 to 399 of CyaA) at the N-terminus of a protein | Euromedex, Souffelweyersheim, France |
| pRJ0202 | Tc^r^ Km^r^ (pSUP202pol4) upstream region of *ecfQ* (EcoRI, PstI) plus PstI fragment of pBSL86 containing Km^r^ cassette (*aphII*) plus downstream region of *ecfQ* (PstI, BamHI) | This work |
| pRJ0203 | Km^r^ (pK18*mobsacB*) carrying upstream region (HindIII, PstI) plus downstream region of blr3042 (PstI, XbaI) | This work |
| pRJ0211 | Ap^r^ (pBluescript SK(+)) containing promoter region of *ecfQ* (EcoRV) | This work |
| pRJ9685 | Tc^r^ (pSUP202pol4) upstream region (EcoRI, PstI) plus downstream region of *osrA* (PstI, XbaI) | This work |
| pRJ9688 | Tc^r^ Km^r^ (pRJ9685) with PstI fragment of pBSL86 containing Km^r^ cassette (*aphII*) | This work |
| pRJ9692 | Tc^r^ Km^r^ ( pSUP202pol4) upstream region of *osrA* (EcoRI, PstI) plus PstI fragment of pBSL86 containing Km^r^ cassette (*aphII*) plus downstream region of *osrA* (PstI, XbaI) | This work |
| pRJ9715 | Tc^r^ Str^r^ (pRJ9685) with PstI fragment of pBSL15-Ω containing Ω cassette (Sp^r^/Str^r^) | This work |
| pRJ9724 | Ap^r^ (pGEM-T Easy) containing *ecfF*-*osrA* including promoter region | This work |
| pRJ9729 | Tc^r^ (pSUP202pol4) 3`-end of *ecfF* plus wild-type version of *osrA* (PstI, XbaI) | This work |
| pRJ9730 | Tc^r^ (pSUP202pol4) 3`-end of bll3040 (PstI, XbaI) | This work |
| pRJ9736 | Tc^r^ (pSUP202pol4) 3`-end of *ecfF* plus *osrA* with codons 129 and 179 mutated to TCC (resulting in OsrA C129S+ C179S) (PstI, XbaI) | This work |
| pRJ9737 | Tc^r^ (pSUP202pol4) 3`- end of *ecfF* and *osrA* with codon 179 mutated to TCC (resulting in OsrA C179S) (PstI, XbaI) | This work |
| pRJ9738 | Tc^r^ (pSUP202pol4) 3`- end of *ecfF* and *osrA* with codon 129 mutated to TCC (resulting in OsrA C129S) (PstI, XbaI) | This work |
| pRJ9744 | Km^r^ (pKT25) encodes fusion of T25 at the N-terminus of wild-type OsrA (EcoRI, PstI) | This work |
| pRJ9746 | Ap^r^ (pUT18C) encodes fusion of T18 at the N-terminus of wild-type EcfF (XbaI, PstI) | This work |
| pRJ9752 | Km^r^ (pKT25) encodes fusion of T25 at the N-terminus of OsrA C129S+C179S (EcoRI, PstI) | This work |
| pRJ9753 | Km^r^ (pKT25) encodes fusion of T25 at the N-terminus of OsrA C179S (EcoRI, PstI) | This work |
| pRJ9754 | Km^r^ (pKT25) encodes fusion of T25 at the N-terminus of OsrA C129S (EcoRI, PstI) | This work |

Alexeyev MF (1995) Three kanamycin resistance gene cassettes with different polylinkers. Biotechniques 18: 52, 54, 56.

Fischer HM, Babst M, Kaspar T, Acuña G, Arigoni F, et al. (1993) One member of a *groESL*-like chaperonin multigene family in *Bradyrhizobium japonicum* is co-regulated with symbiotic nitrogen fixation genes. EMBO J 12: 2901-2912.

Lindemann A, Koch M, Pessi G, Müller AJ, Balsiger S, et al. (2010) Host-specific symbiotic requirement of BdeAB, a RegR-controlled RND-type efflux system in *Bradyrhizobium japonicum*. FEMS Microbiol Lett 312: 184-191.

Regensburger B, Hennecke H (1983) RNA polymerase from *Rhizobium japonicum*. Arch Microbiol 135: 103-109.

Schäfer A, Tauch A, Jäger W, Kalinowski J, Thierbach G, et al. (1994) Small mobilizable multi-purpose cloning vectors derived from the *Escherichia coli* plasmids pK18 and pK19: selection of defined deletions in the chromosome of *Corynebacterium glutamicum*. Gene 145: 69-73.

Simon R, Priefer U, Pühler A (1983) A broad host range mobilization system for *in vivo* genetic-engineering - transposon mutagenesis in gram-negative bacteria. Bio/Technology 1: 784-791.
