# Supplementary material for: Reactive Oxygen Species-Inducible ECF σ Factors of Bradyrhizobium japonicum
Source: PLoS One. 2012 Aug 16;7(8):e43421. doi: 10.1371/journal.pone.0043421 (PMC3420878; doi:10.1371/journal.pone.0043421)
Supplement: Table S6 — Primers used in this study. (DOCX) [file pone.0043421.s008.docx]

**Table S6.** Primers used in this study.

| **Primer pairs** | **Sequence^a^** | **Resulting PCR products or location** | **Used for** |
| --- | --- | --- | --- |
| up-1028-F  up-1028-R | GCGCGAATTCCGAACTGGCCAATGC  GCGCGGATCCTTCCTTGGACCAAAGTG | 593 bp upstream region of *ecfQ* | pRJ0202 |
| down-1028-F  down-1028-R | GCAAGGATCCAGCGGCCCTATTCCC  GAAGCGGCCGCACGTTCGGATCGAAGTC | 684 bp downstream region of *ecfQ* | pRJ0202 |
| up-3042-F  up-3042-R | CCTTAAGCTTGATCGTGCCGTCATAG  CCAACTGCAGAACTTACCGCAGTCATAC | 588 bp upstream region of blr3042 | pRJ0203 |
| down-3042-F  down-3042-R | CCAACTGCAGCTGGCCGAACTGCTGAAG  CCAATCTAGACGCGCTGGTCGAAAG | 514 bp downstream region of blr3042 | pRJ0203 |
| up-3038-F  up-3038-R | GAATTCGTCGAGATCGTTGAGCTGGTCG  CTGCAGCATGCAATTCGGCCGCTCTTTC | 778 bp upstream region of *ecfF* | pRJ9688,  pRJ9715 |
| up-3039-F  up-3039-R | GAATTCGACGCCGTGGCGTGACGATA  CTGCAGGCCGCAAGCGAGCGAATGAG | 842 bp upstream region of osrA | pRJ9692 |
| down-3039-F  down-3039-R | CTGCAGTAGCCGCGATCGGAGCGCTG  TCTAGAGGTGATCGAGGTCGCAGGAC | 875 bp downstream region of *osrA* | pRJ9688,  pRJ9692,  pRJ9715 |
| 3039-2G-F1  3039-2G-R1 | ATACTGCAGGGATGGATACCGATCA  ATAGAATTCTCTAATACCGCAACAC | 660 bp coding region of *osrA* | pRJ9744,  pRJ9752-54 |
| 3038-2G-F1  3038-2G-R1 | AATCTGCAGGATGATGAGGGCGCGGGT  ATATCTAGACTAGTGGTCCCGCAGTTTG | 580 bp coding region of *ecfF* | pRJ9746 |
| 3039Compl-F4  3039Compl-R4 | CATCTAGAGCAGTCACGCCGGTGCT  GACTGCAGGCCTAATACCGCAACACC | 1116 bp 3`end of *ecfF* and *osrA* | pRJ9729,  pRJ9736-38 |
| ComplC-F4  ComplC-R4 | CATCTAGAATTAGGCCGCGGGCGT  GACTGCAGTCGAGCGTGGTCCAGGAAG | 451 bp 3`end of bll3040 | pRJ9730 |
| 1028-RT-F  1028-RT-R | GAGATCATCACCCTCGTCTACTAC  CATAGAACATCCGCGTCTTCAC | 100 bp internal region of *ecfQ* | qRT-PCR |
| 3038-RT-F  3038-RT-R | CGTATCATCGCCTGTTGAAG  GCCAACAGAATCTCCTGCAC | 121 bp internal region of *ecfF* | qRT-PCR |
| 1028-seq-F  1028-seq-R | GAATGAATTCGATCCGGGACCCATAGC  ATATGGATCCCGCAGGATGAAGCGGTA | 584 bp upstream region of *ecfQ* and 5’ end of *ecfQ* | pRJ0211 |
| 3089Compl-F3  3089Compl-R3 | CATCTAGAAGAGTGACGCCGGTGCT  GACTGCAGCCTAATACCGCAACACCC | 1657 bp upstream region of *ecfF*, *ecfF* and *osrA* | pRJ9724 |
| pe-1028-1  pe-1028-2 | CCAGAAGCATATCGTCCGAAGTG  ACGTCCGGTTGCCGTCGGCAATGC | located in *ecfQ* | primer extension |
| pe-3038-1 | GCGTGACTGCCTTCAACA | located in *ecfF* |  |

^a^ Engineered restriction enzyme sites are underlined.
